# Dynamic histone acetylation in floral volatile synthesis and emission in petunia flowers

**DOI:** 10.1093/jxb/erab072

**Published:** 2021-02-19

**Authors:** Ryan M Patrick, Xing-Qi Huang, Natalia Dudareva, Ying Li

**Affiliations:** 1 Department of Horticulture and Landscape Architecture, Purdue University, West Lafayette, IN 47907,USA; 2 Purdue Center for Plant Biology, Purdue University, West Lafayette, IN 47907,USA; 3 Department of Biochemistry, Purdue University, West Lafayette, IN 47907,USA; 4 Oklahoma State University, USA

**Keywords:** Epigenome, floral volatile, histone acetylation, organic compounds, secondary metabolites

## Abstract

Biosynthesis of secondary metabolites relies on primary metabolic pathways to provide precursors, energy, and cofactors, thus requiring coordinated regulation of primary and secondary metabolic networks. However, to date, it remains largely unknown how this coordination is achieved. Using *Petunia hybrida* flowers, which emit high levels of phenylpropanoid/benzenoid volatile organic compounds (VOCs), we uncovered genome-wide dynamic deposition of histone H3 lysine 9 acetylation (H3K9ac) during anthesis as an underlying mechanism to coordinate primary and secondary metabolic networks. The observed epigenome reprogramming is accompanied by transcriptional activation at gene loci involved in primary metabolic pathways that provide precursor phenylalanine, as well as secondary metabolic pathways to produce volatile compounds. We also observed transcriptional repression among genes involved in alternative phenylpropanoid branches that compete for metabolic precursors. We show that GNAT family histone acetyltransferase(s) (HATs) are required for the expression of genes involved in VOC biosynthesis and emission, by using chemical inhibitors of HATs, and by knocking down a specific HAT gene, *ELP3*, through transient RNAi. Together, our study supports that regulatory mechanisms at chromatin level may play an essential role in activating primary and secondary metabolic pathways to regulate VOC synthesis in petunia flowers.

## Teaser

Our study shows that post-translational modification of histones is essential for regulating the biosynthesis and emission of floral scent compounds, thus providing insights into the regulation of secondary metabolism at chromatin level.

## Introduction

Plants have a remarkable ability to synthesize a vast array of secondary metabolites that are not only vital for plant growth, development, reproduction, and defense, but also play crucial roles for mankind in food, medicine, industrial raw materials, and biofuels. A unique subset of secondary metabolites consists of volatile organic compounds (VOCs) that plants use for communication and interaction with the surrounding environment (Muhlemann *et al.*, 2014*a*). Structurally diverse VOCs are released from every plant tissue and play key roles in attracting pollinators and seed dispersers (Borges *et al.*, 2008; Brodmann *et al.*, 2009; Byers *et al.*, 2014), above- and below-ground herbivore defense (Kessler and Baldwin, 2001; Kost and Heil, 2006), protection from pathogens and abiotic stresses (Affek and Yakir, 2002; Yi *et al.*, 2009; Ameye *et al.*, 2015), and plant–plant (Engelberth *et al.*, 2004; Kost and Heil, 2006) and inter-organ signaling (Boachon *et al.*, 2019). In most cases, emitted VOCs are comprised of complex blends of metabolites; for example, there are up to 100 different compounds in floral scent bouquets (Dudareva *et al.*, 2006). Synthesis and release of such complex mixtures require orchestrated activation of multiple enzymatic steps among and within biosynthetic pathways.

In the last two decades, significant progress has been made in the isolation and characterization of genes responsible for the formation of plant VOCs. In general, biosynthesis of secondary metabolites relies on primary metabolic pathways, which provide precursors for their formation. It has been shown that volatiles are synthesized *de novo* in the tissues from which they are emitted, and their production and emission are spatially (Dudareva *et al.*, 1996; Kolosova *et al.*, 2001*a*; Underwood *et al.*, 2005), developmentally, and/or temporally regulated (Negre *et al.*, 2003; Colquhoun *et al.*, 2010; Spitzer-Rimon *et al.*, 2010; Fenske *et al.*, 2015), and depend on biotic and abiotic factors (Qualley and Dudareva, 2008; Dudareva *et al.*, 2013). VOC biosynthesis is mainly regulated at the level of gene expression (Dudareva *et al.*, 1996, 2000; Gershenzon *et al.*, 2000; Muhlemann *et al.*, 2012), by the activity of enzymes responsible for the final step of VOC formation, and substrate availability (De Moraes *et al.*, 2001; Martin *et al.*, 2003; Arimura *et al.*, 2004; Effmert *et al.*, 2005; Guterman *et al.*, 2006; Vogel *et al.*, 2008; Colquhoun *et al.*, 2010; Maeda *et al.*, 2010). Genes involved in the formation of VOCs have been shown to exhibit coordinated transcriptional activation coinciding with the VOC emission (Dudareva *et al.*, 2003; Verdonk *et al.*, 2005; Colquhoun *et al.*, 2010). However, to date, it has still remained largely unknown how the changes in the expression status are achieved and the regulatory mechanisms underlying this transcriptional reprogramming.

It has become increasingly clear that epigenetic regulation, including histone modification, DNA methylation, and chromatin remodeling, significantly contributes to transcriptional regulation during plant development and environmental responses (Chinnusamy and Zhu, 2009; He *et al.*, 2011). Histone N-terminal tails are dynamically and reversibly modified with chemical groups and small proteins by histone-modifying enzymes (Zhang and Reinberg, 2001). These modifications can alter the local chromatin landscape of a gene locus and are recognized by structural and regulatory proteins, which leads to transcriptional activation or silencing of the gene (Rea *et al.*, 2000; Strahl and Allis, 2000; Musselman *et al.*, 2012). In fungi, histone modification was shown to control gene clusters governing production of secondary metabolites (Strauss and Reyes-Dominguez, 2011; Pfannenstiel and Keller, 2019). However, our understanding of the chromatin-level regulation of secondary metabolic pathways in plants remains rather limited.

*Petunia hybrida* cv. Mitchell flowers emit high levels of predominantly phenylalanine-derived phenylpropanoid/benzenoid volatiles, and subsequently have been widely used as a model system (Schuurink *et al.*, 2006; Dudareva *et al.*, 2013) to identify and characterize enzymes and genes involved not only in the biosynthesis and emission of scent compounds, but also in the formation of their precursor, phenylalanine (Maeda and Dudareva, 2012). In this study, we use petunia flowers to investigate chromatin-level mechanisms that regulate genes involved in the formation of primary metabolite precursors and secondary metabolite volatile compounds, at a genome-wide level using ChIP sequencing (ChIP-Seq). We show that chromatin modifications during anthesis, specifically H3K9ac, facilitate the activation of the shikimate and phenylalanine synthetic pathway to provide the primary metabolite precursor, as well as distinct secondary metabolic pathways to generate the floral VOC products.

## Materials and methods

### Chromatin immunoprecipitation

Approximately 2 g of corolla tissue from *P. hybrida* cv. Mitchell flowers grown under standard greenhouse conditions were harvested at the day 0 (bud) or day 2 (post-anthesis) stage at 15.00 h in three replicates. The corolla tissue was fixed in 37 ml of 1% formaldehyde under vacuum twice, first for 15 min and then for 10 min. The reaction was stopped with addition of 2.5 ml of 2 M glycine, placed under vacuum for 5 min, and then washed three times with ultrapure water. The fixed tissue was then dried and frozen in liquid nitrogen. Chromatin extraction and immunoprecipitation were performed as previously described (Gendrel *et al.*, 2005; Li *et al.*, 2015). In detail, frozen, ground tissue was suspended in 30 ml of extraction buffer (EB1), which was filtered through Miracloth (Millipore Sigma) and then centrifuged at 4 °C (1400 *g*, 15 min). The pellet was resuspended in 1 ml of a second extraction buffer (EB2) and centrifuged again at 4 °C (11 000 *g*, 15 min). The pellet was then resuspended in 300 μl of a third extraction buffer (EB3) and overlaid on 300 μl of EB3 and centrifuged at 4 °C (16 000 *g*, 1 h) to obtain the chromatin pellet. The pellet was resuspended in 300 μl of nuclei lysis buffer and sonicated with a BioRuptor Pico sonicator (Diagenode) for 26–36 cycles to obtain an ~200 bp fragment distribution. Cell debris was pelleted at 4 °C (12 000 *g*, 5 min) and separated from the supernatant containing chromatin. A 20 μl aliquot of the supernatant was kept as input DNA and the rest was diluted with ChIP dilution buffer for immunoprecipitation. To perform immunoprecipitation, Protein A Dynabeads (Invitrogen) were first resuspended in ChIP dilution buffer. A 50 μl aliquot of beads was used for pre-clearance of chromatin (rotating for 2 h at 4 °C) and, during this time, a second 50 μl of beads were incubated with antibody. Anti-H3K4me3 (07-473, MilliporeSigma) and anti-H3K9ac (07-352, MilliporeSigma) antibodies were used for immunoprecipitation. Pre-cleared chromatin was then added to antibody-bound beads and incubated with rotation overnight at 4 °C. Beads were washed twice each with a series of buffers: low salt wash buffer, high salt wash buffer, LiCl wash buffer, and Tris–EDTA. Chromatin was then eluted twice with 250 μl of elution buffer at 65 °C for 15 min. The two eluates were combined. The DNA cross-linking was reversed with addition of 20 μl of 5 M NaCl and incubation at 65 °C overnight. A 1 μl aliquot of proteinase K (Invitrogen) was added to digest the proteins at 45 °C for 1 h, then an equal volume of 25:24:1 phenol/chloroform/isoamyl alcohol, pH 8.0 (Invitrogen) was added to phase separate the DNA and the proteins. The DNA in the aqueous phase was precipitated with sodium acetate and ethanol along with 1 μl of glycogen carrier (Thermo Scientific) overnight at –20 °C and then centrifuged to pellet at 4 °C (12 000 *g*, 10 min). The pellet was washed with cold 70% ethanol, dried, and then resuspended in water. The concentration was measured using a Qubit dsDNA High Sensitivity assay kit (Invitrogen).

### ChIP sequencing and data analysis

A 10 ng aliquot of input or immunoprecipitated DNA in three replicates per condition were used in library preparation for dual index Illumina paired-end sequencing (Para *et al.*, 2018) with the following modifications: (i) using NEBNext Multiplex Oligos (New England Biolabs) for adaptor ligation and barcoding primers, following the manufacturer’s protocols; (ii) using AMPure XP beads (Beckman Coulter) for size selection; and (iii) using 18 cycles of PCR amplification with Phusion High Fidelity DNA polymerase (New England Biolabs). Libraries were pooled and sequenced (paired-end 2×150 bp) on an Illumina NovaSeq 6000 at the Purdue Genomics Core Facility. Reads were trimmed for adaptors with cutadapt (Martin, 2011) and aligned to the *Petunia axillaris* (v1.6.2) and *P. inflata* (v1.0.1) genomes (Fernandez-Pozo *et al.*, 2015*a*; Bombarely *et al.*, 2016) with Bowtie 2 (Langmead and Salzberg, 2012). Properly paired read alignments were then converted to bed format representing each insert fragment using bedtools (Quinlan and Hall, 2010). The aligned fragments were then used to call differential enrichment of sequencing peaks for H3K4me3 and H3K9ac ChIP between day 2 and day 0 using the corresponding input DNA library as a background. For peak calling, SICER-df.sh (Zang *et al.*, 2009; Xu *et al.*, 2014) was run for all *Petunia* scaffolds containing at least one annotated gene, with a gap size of 200, a window size of 200, an effective genome size of 0.9, and with a false discovery rate (FDR) <0.01 cut-off for significance. Significantly increased or decreased peaks at a ≥2-fold were intersected with annotated petunia gene features using bedtools (Quinlan and Hall, 2010). Genes which overlapped with significant peaks in all three replicates were considered significant differentially modified genes (DMGs). For downstream analyses of gene set enrichment, *P. axillaris* gene features were used as a background. The significance of overlapping gene sets between ChIP-Seq and RNA-Seq analyses was evaluated by hypergeometric test using the phyper function in R. Integrative Genomics Viewer (IGV) was used for visualization of ChIP-Seq data (Robinson *et al.*, 2011).

### RNA sequencing analysis

RNA sequencing data for day 0 and day 2 petunia corolla tissue were obtained from the NBCI Gene Expression Omnibus (GSE70948) (Widhalm *et al.*, 2015). Reads were trimmed with an in-house Python script and aligned to the *P. axillaris* (v1.6.2) and *P. inflata* (v1.0.1) genomes (Fernandez-Pozo *et al.*, 2015*a*; Bombarely *et al.*, 2016) using TopHat2 (Kim *et al.*, 2013). Aligned reads were assigned to petunia genes with HTSeq (Anders *et al.*, 2015). Differentially expressed genes (DEGs) were determined using DESeq2 (Love *et al.*, 2014) with cut-offs of |log2 fold change| >1 and an FDR <0.05.

### Gene Ontology enrichment analysis

In order to determine enriched Gene Ontology (GO) terms in gene sets of interest in petunia, the best *Arabidopsis thaliana* homolog to each *P. axillaris* protein was determined using blastp against the Araport11 protein database (Cheng *et al.*, 2017) with an E-value cut-off of 1.0e-08 by the BLAST+ toolkit (Camacho *et al.*, 2009). GO term annotations for *A. thaliana* genes curated by TAIR (Berardini *et al.*, 2004) were then assigned to the *P. axillaris* homologs to populate a GO term background for petunia. The significant enrichment of GO terms in gene sets of interest versus the background were detected using an in-house Python script, where a hypergeometric test (SciPy stats library, https://www.scipy.org) was used to calculate statistical significance of enrichment, and FDR correction (Benjamini and Hochberg, 1995) was applied to control for multitesting error using the Python module ‘statsmodels’ (Seabold and Perktold, 2010). REVIGO was used for visualization of enriched GO terms (Supek *et al.*, 2011). Enrichment levels of statistically significant GO terms were used to create heatmaps with the R packages ‘pheatmap’ (https://cran.r-project.org/web/packages/pheatmap/index.html) and ‘RColorBrewer’ (https://cran.r-project.org/web/packages/RColorBrewer/index.html).

### Metabolic pathway analysis

Aromatic amino acid biosynthesis and phenylpropanoid pathway genes in *P. axillaris* were identified based on supplementary notes to the publication of petunia genomes (Bombarely *et al.*, 2016) or collected knowledge of the pathways (Zhao and Dixon, 2011; Maeda and Dudareva, 2012; Dudareva *et al.*, 2013; Muhlemann *et al.*, 2014*b*; Passeri *et al.*, 2016), with members of each pathway identified by BLAST homology. Two putative *CAD* (cinnamyl alcohol dehydrogenase) genes were identified in *P. axillaris* based on homology to the major *CAD* genes contributing to lignin biosynthesis in *A. thaliana* (Sibout *et al.*, 2005) and *Nicotiana attenuata* (Kaur *et al.*, 2012). The petunia CAD1 and CAD2 proteins have 92% and 78% identity with the *N. attenuata* CAD1 protein, respectively. Putative peroxidase and laccase genes involved in lignin polymerization were identified by BLAST homology to genes identified in *A. thaliana* (Zhao *et al.*, 2013). Significance of overlaps between metabolic pathway genes and gene lists from ChIP-Seq and RNA-Seq analyses was evaluated by hypergeometric test in R. Overlaps between gene sets were visualized using Circos (Krzywinski *et al.*, 2009).

### Chemical inhibition assays

For histone acetyltransferase (HAT) inhibitor assays, *P. hybrida* cv. Mitchell flowers grown under standard greenhouse conditions were harvested at the day 0 (bud) stage at 15.00 h and floated in a 3% sucrose solution with mock treatment (0.1% DMSO) or the HAT inhibitor C646 (MilliporeSigma) or MB-3 (MilliporeSigma) at a final concentration of 100 μM in 0.1% DMSO. Treatment with inhibitors did not affect anthesis, as all flowers were open by the afternoon of day 1. Corolla tissue was harvested and frozen in liquid nitrogen 1 h before the end of the light period at 19.00 h in four biological replicates of two flowers each, on day 0, day 1, and day 2. Gibberellic acid inhibitor assays were performed similarly, with uniconazole (Lkt Laboratories) or flurprimidol (bioWORLD) at a final concentration of 100 μM in 0.1% DMSO, with a single end-point harvest at day 2. RNA was extracted with the RNeasy Plant Mini Kit (Qiagen), treated with recombinant RNase-free DNase I (Roche) at 37 °C for 1 h, followed by a clean up with the RNeasy Mini Kit (Qiagen). SuperScript IV (Invitrogen) was used to generate cDNA with an oligo(dT) primer. Quantitative reverse transcription–PCR (qRT–PCR) was performed with PowerUp SYBR Green (Applied Biosystems) using primers designed to specification with Primer3 (Untergasser *et al.*, 2012) with the sequences listed in Supplementary Table S1 or as previously described for *UBQ10* and *PhABCG1* (Adebesin *et al.*, 2017). Relative gene expression was normalized to two reference genes, *FBP1* and *UBQ10*. For ChIP-qPCR to confirm chemical inhibition of HAT activity, flowers were mock treated or treated with MB-3 as above, and at day 2 were used for ChIP as described above for ChIP-Seq, with anti-H3K9ac (07-352, Millipore Sigma) and anti-H3K14ac (07-353, Millipore Sigma) antibodies used for immunoprecipitation. Primers designed to gene body acetylation peaks (Supplementary Table S1) were used for qPCR with PowerUp SYBR Green (Applied Biosystems) in order to determine the pull-down percentage relative to input, with normalization to housekeeping genes *ACTIN* (Peaxi162Scf00583g00529) and *PP2AA3* (Peaxi162Scf00382g00122).

### ELP3 down-regulation through transient RNAi

A 300 bp sequence specific for RNAi-mediated silencing of ELP3 was chosen using the Sol Genomics Network VIGS (virus-induced gene silencing) tool (Fernandez-Pozo *et al.*, 2015*b*) and then amplified from *P. hybrida* cDNA using primers including an *attB* recombination site. The fragment was then cloned into the pDONR/Zeo gateway vector (Life Technologies) following the manufacturer’s instructions and subcloned into the destination vector pK7GWIWG2(II) in a sense and antisense direction to facilitate hairpin formation by Invitrogen Gateway LR Clonase II Enzyme mix (Life Technologies). Binary vectors were transformed into *Agrobacterium tumefaciens* strain GV3101 with freeze–thaw cycles. Transient *ELP3* down-regulation was achieved similarly to as previously described (Yoo *et al.*, 2013) using vacuum infiltration of at least 18 petunia flowers at the day 1 stage, with transformed *Agrobacterium* containing pK7GWIWG2(II)-ELP3 or pK7GWIWG2(II) empty vector control at an OD_600_ of 0.8. Infiltrated flowers were kept in 5% sucrose solution in the dark for an additional 48 h before floral scent was collected for 4 h from 17.00 h to 21.00 h. Following scent collection, total RNA was extracted from petunia flowers using the Spectrum Plant Total RNA Kit (Sigma-Aldrich). About 1 μg of total RNA was reverse transcribed to first-strand cDNA in a 10 μl reaction using the EasyScript cDNA synthesis kit (Applied Biological Materials) and used for qRT–PCR as described above.

### Targeted metabolite profiling

Petunia volatiles were collected from infiltrated flowers by a closed-loop stripping method and analyzed by GC-MS (Widhalm *et al.*, 2015). Briefly, floral scents were absorbed in VOC collection traps containing 20 mg of Porapak Q (80–100 mesh) matrix (Waters). VOCs were eluted from scent collection traps with 200 μl of dichloromethane. A 1 μg aliquot of naphthalene was added to the eluted sample as an internal standard. Samples were analyzed on an Agilent 6890N-5975B GC-MS system. Quantitation of different volatile compounds was performed based on standard curves generated with commercially available standards.

## Results

### Coordinated chromatin modification and transcriptomic reprogramming in petunia corolla during flower development

In petunia flowers, floral VOCs are produced primarily in the corolla. The levels of phenylalanine precursor and VOCs are developmentally co-regulated during the life span of the flower, achieving a maximum on the second day post-anthesis (Verdonk *et al.*, 2005; Maeda *et al.*, 2010). We hypothesized that a genome-wide reprogramming of histone modifications occurs in the corolla during floral development to promote active transcription of genes involved in VOC synthesis, including the primary and secondary metabolite pathways. To test this hypothesis, corolla tissues of *P. hybrida* cv. Mitchell were collected for ChIP-Seq in three biological replicates, at two time points representing the lowest and highest VOC production: immediately pre-anthesis buds (day 0) and open flowers (day 2). As activation of VOC pathway genes was of particular interest, histone marks associated with transcriptional activation, H3K9ac and H3K4me3, were chosen as the focus of this study (Ha *et al.*, 2011; Du *et al.*, 2013). The ChIP-Seq reads were generated using the Illumina NovaSeq platform and then mapped to parental genomes of *P. hybrida*, *P. axillaris*, and *P. inflata* (Bombarely *et al.*, 2016). The majority reads mapped to the *P. axillaris* genome (Supplementary Fig. S1), which agrees with previous observations (Bombarely *et al.*, 2016). Therefore, *P. axillaris* gene set were used for downstream bioinformatic analysis to provide an appropriate statistical background. Genes associated with significantly increased or decreased levels of H3K9ac or H3K4me3 on day 2 relative to day 0 in all three biological replicates were determined using SICER (FDR <0.01, fold change >2) (Zang *et al.*, 2009) and referred to as DMGs.

**Fig. 1.**
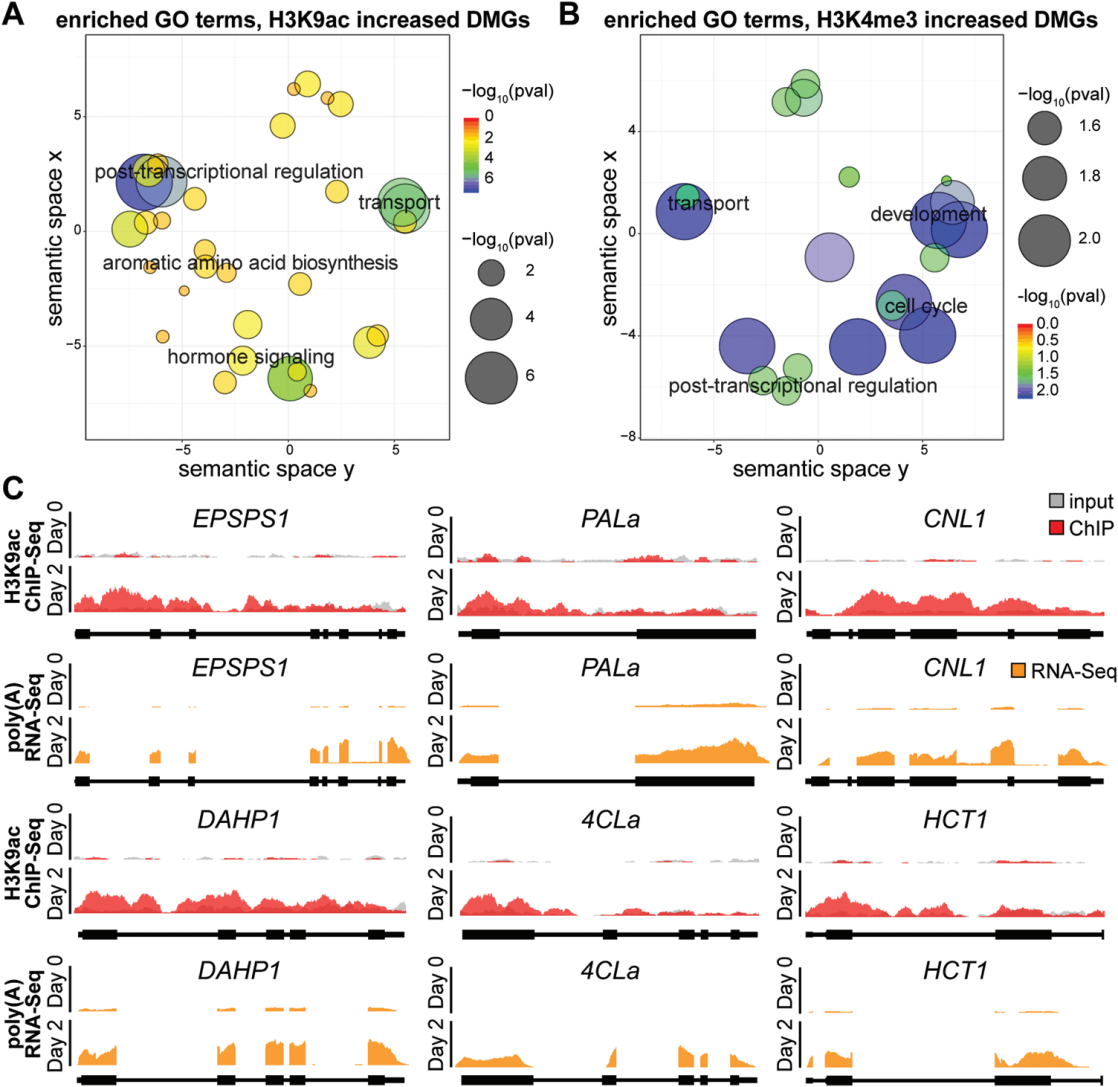
Dynamic regulation of histone modifications during anthesis in petunia corolla. Significant GO terms enriched among DMGs with increased H3K9ac (A) or increased H3K4me3 (B) on day 2 versus day 0 in petunia corolla are shown in semantic spaces generated by REVIGO. Each circle in the semantic space represents a significant GO term, and the size and color of the circle represent the level of significance of the enrichment. (C) Histone ChIP-Seq and RNA-Seq coverage showing dynamic H3K9ac deposition along the gene body of specific VOC genes from day 0 to day 2 and coincident increase in mRNA level. The sequencing depth was scaled to library size. Representative results from one of three independent biological replicates are shown. 4CL, 4-coumaryl-CoA ligase; CNL, cinnamoyl-CoA ligase; DAHP, 3-deoxy-d-*arabino*-heptulosonate 7-phosphate synthase; EPSPS, 5-*enol*pyruvylshikimate 3-phosphate synthase; HCT, hydroxycinnamoyl-CoA:shikimate/quinate hydroxycinnamoyl transferase; PAL, phenylalanine ammonia lyase.

Overall, we identified a widespread reprogramming of histone modifications in petunia corolla from the bud stage (day 0) to open flower (day 2), in the form of increased levels of H3K9ac or H3K4me3 at hundreds to thousands of gene loci. Specifically, 4207 DMGs exhibit increased H3K9ac from day 0 to day 2, and 355 DMGs displayed decreased H3K9ac (Supplementary Dataset S1). For H3K4me3, we identified 855 DMGs with increased H3K4me3 and nine DMGs with reduced H3K4me3 (Supplementary Dataset S1). To determine whether specific biological processes are associated with these DMGs, we developed an in-house GO enrichment analysis pipeline for petunia and identified GO terms significantly enriched (FDR <0.05) among DMGs (Fig. 1A, B; Supplementary Tables S2, S3). Interestingly, DMGs with increased H3K9ac were uniquely enriched with ‘aromatic amino acid biosynthesis’ (Fig. 1A; Supplementary Table S2), suggesting that H3K9ac, but not H3K4me3, is specifically involved in activating phenylalanine biosynthesis to provide precursor for floral VOCs. Indeed, we observed a significant increase of H3K9ac levels from day 0 to day 2 at gene loci involved in the shikimate pathway and the general phenylpropanoid pathway that synthesize the precursor metabolites phenylalanine and phenylalanine derivatives for VOC formation (Fig. 1C: *EPSPS1*, *DAHP1*, *PALa*, and *4CLa*). Furthermore, we observed increased H3K9ac at gene loci involved in the formation of VOCs including volatile benzenoid/phenylpropanoid (Fig. 1C; *CNL*) and volatile phenylpropene (Fig. 1C; *HCT*). In addition, DMGs with increased H3K9ac were enriched with GO terms related to post-transcriptional regulation, hormone signaling, and intracellular protein transport, while DMGs with increased H3K4me3 were enriched with GO terms for post-transcriptional regulation and transport, and tissue development and the cell cycle (Fig. 1A, B; Supplementary Tables S2, S3). There was no significant GO term enriched among genes showing decreased levels of H3K9ac or H3K4me3.

**Fig. 2.**
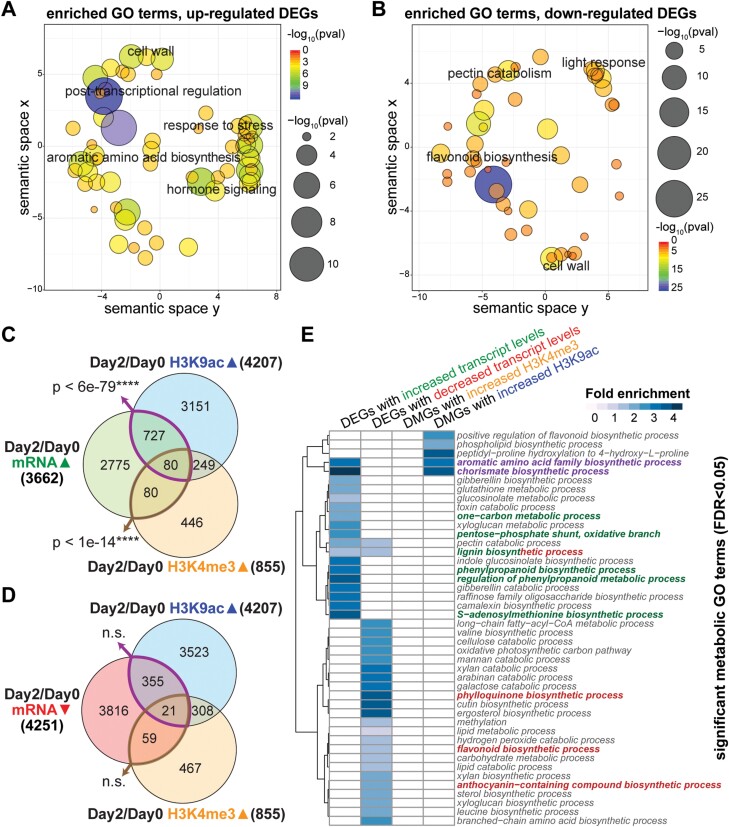
Transcriptionally and epigenetically regulated genes and metabolic pathways during anthesis in petunia corolla. Significant GO terms enriched among DEGs with increased transcript levels (A) or decreased transcript levels (B) on day 2 versus day 0 in petunia corolla are shown in semantic spaces generated by REVIGO. Each circle in the semantic space represents a significant GO term, and the size and color of the circle represent the level of significance of the enrichment. Overlaps between sets of DMGs with increased H3K9ac or H3K4me3 levels and DEGs with increased (C) or decreased (D) transcript levels are shown in Venn diagrams. The *P*-value for the intersection between gene sets was determined by hypergeometric test. (E) Heatmap depicting the fold enrichment of metabolic GO terms significantly enriched among the DEGs or DMGs. White color represents a lack of significant enrichment. Phenylpropanoid and VOC synthesis-related metabolic terms are highlighted in green for up-regulated processes, red for down-regulated processes, and purple for biological processes regulated at both the transcript and chromatin levels.

To understand the effects of observed histone modification patterns on gene expression, RNA-Seq data generated from the same tissue and the same developmental stages as the ChIP-Seq were analyzed (Widhalm *et al.*, 2015). We found a global reprogramming of gene expression in the corolla from day 0 to day 2, including 3662 DEGs that were up-regulated and 4251 that were down-regulated, determined by DESeq2 with a cut-off of FDR <0.05 and fold change >2 (Love *et al.*, 2014) (Supplementary Dataset S2; Supplementary Fig. S2). As expected, the six representative phenylalanine and VOC biosynthetic genes in Fig. 1C also showed significantly increased mRNA levels (Fig. 1C), suggesting that the hyperacetylation of H3K9ac is associated with increased expression of these genes. Indeed, aromatic amino acid biosynthesis was enriched among up-regulated DEGs, while flavonoid and anthocyanin biosynthesis were enriched among down-regulated DEGs; interestingly, both up- and down-regulated DEGs were significantly enriched with genes involved in cell wall modification (Fig. 2A, B; Supplementary Table S4, explained in detail later). In addition, up-regulated DEGs were enriched with genes involved in post-transcriptional regulation, stress response, and hormone signaling (Fig. 2A; Supplementary Table S4), while down-regulated DEGs were enriched with genes involved in light response (Fig. 2B; Supplementary Table S4).

To investigate the extent to which changes of histone modifications at a gene locus co-occur with changes in transcript level, the DMGs identified by ChIP-Seq were compared with DEGs determined by RNA-Seq. Significant overlaps were observed between DEGs with increased transcript levels and DMGs that gained either H3K9ac or H3K4me3 on day 2 versus day 0 (Fig. 2C). In contrast, genes that gain H3K9ac or H3K4me3 have no significant overlap with down-regulated DEGs (Fig. 2D). Genes associated with reduced H3K9ac did not show significant overlap with either set of DEGs (Supplementary Fig. S3). Overall, our results indicate that increased H3K9ac and H3K4me3 are associated with gene activation during anthesis in petunia flowers.

### Transcriptional and epigenetic regulation of phenylpropanoid metabolism in petunia flowers

To further investigate the regulation of biosynthesis and emission of floral VOCs, we first examined in detail the enriched metabolic GO terms among DEGs and DMGs for their relevance to floral VOC emission (Fig. 2E). Interestingly, only two significantly over-represented GO terms were found to be shared between DMGs (with increased H3K9ac) and DEGs (up-regulated): ‘chorismate biosynthetic process’ and ‘aromatic amino acid family metabolic process’ (Fig. 2E, GO terms in purple). Increased flux through the shikimate pathway to promote production of chorismate and subsequently phenylalanine, the predominant precursor of petunia floral VOCs, is known to be an important factor contributing toward VOC production (Schuurink *et al.*, 2006; Colquhoun *et al.*, 2010; Oliva *et al.*, 2015; Widhalm *et al.*, 2015). Our integrated analysis now revealed that this crucial process is regulated at both epigenetic and transcript levels.

In addition to the production of the precursors chorismate and phenylalanine, more significant GO terms relevant to floral VOC synthesis were identified among the up-regulated DEGs, including ‘phenylpropanoid biosynthetic process’ and ‘regulation of phenylpropanoid metabolic process’ (highlighted in green in Fig. 2E). The term ‘pentose-phosphate shunt, oxidative branch’ is also of relevance, as the pentose phosphate pathway provides the erythrose 4-phosphate (E4P) precursor for the shikimate pathway to produce chorismate and subsequently phenylalanine (Maeda and Dudareva, 2012). Also present as enriched GO terms are ‘*S*-adenosylmethionine (SAM) biosynthetic process’ and the connected ‘one-carbon metabolic process’. SAM is a methyl donor in the production of volatile metabolites such as methylbenzoate and eugenol (Kolosova *et al.*, 2001*b*; Shaipulah *et al.*, 2016). It has been shown that *SAM SYNTHETASE* is regulated coordinately with VOC and shikimate pathway enzymes in petunia during flower development (Verdonk *et al.*, 2003, 2005). Here, our transcriptome analysis expanded the previous reports to the genome-wide level, revealing that multiple genes in the SAM biosynthetic pathway are targets of transcriptional activation during corolla maturation and VOC synthesis.

GO term analysis of down-regulated DEGs revealed transcriptional repression of metabolic pathways that compete with VOC metabolism for precursors or cofactors, including ‘flavonoid biosynthetic process’, ‘anthocyanin-containing compound biosynthetic process’, and ‘phylloquinone biosynthetic process’ (Fig. 2E, terms highlighted in red). Phylloquinone is produced from chorismate, while flavonoids and anthocyanins are derivatives of phenylalanine and share metabolic intermediates with VOC pathways (Maeda and Dudareva, 2012; Bombarely *et al.*, 2016). Production of high levels of VOCs in *P. hybrida* flowers is therefore coincident with the reduced expression of genes involved in competing pathways that consume phenylpropanoid metabolic precursors. Interestingly, the GO term for ‘lignin biosynthetic process’ is present among up-regulated DEGs as well as down-regulated DEGs (Fig. 2E).

Overall, our analysis of GO terms revealed coordinated H3K9ac deposition at gene loci involved in the chorismate and aromatic amino acid metabolic pathways in petunia flowers, accompanied by transcriptional activation of these pathways. We also showed that global transcriptional reprogramming deactivates phenylpropanoid metabolic branches that would draw phenylalanine or other phenylpropanoid intermediates away from VOC production such as anthocyanin/flavonoid biosynthesis pathways. However, these enrichment analyses are based on GO annotations propagated from the Arabidopsis model, thus lineage-specific secondary metabolism genes involved in floral VOC synthesis and emission are overlooked. Moreover, we were interested in identifying the specific points of transcriptional and chromatin regulation within primary and secondary metabolic networks. Therefore, we further examined in detail individual metabolic branches and pathways related to VOC metabolism for transcriptional and epigenetic regulatory targets, based on an extensive collection of curated knowledge of these pathways in petunia (Supplementary Tables S5–S7), as detailed below.

#### Shikimate and phenylalanine biosynthesis pathways

**Fig. 3.**
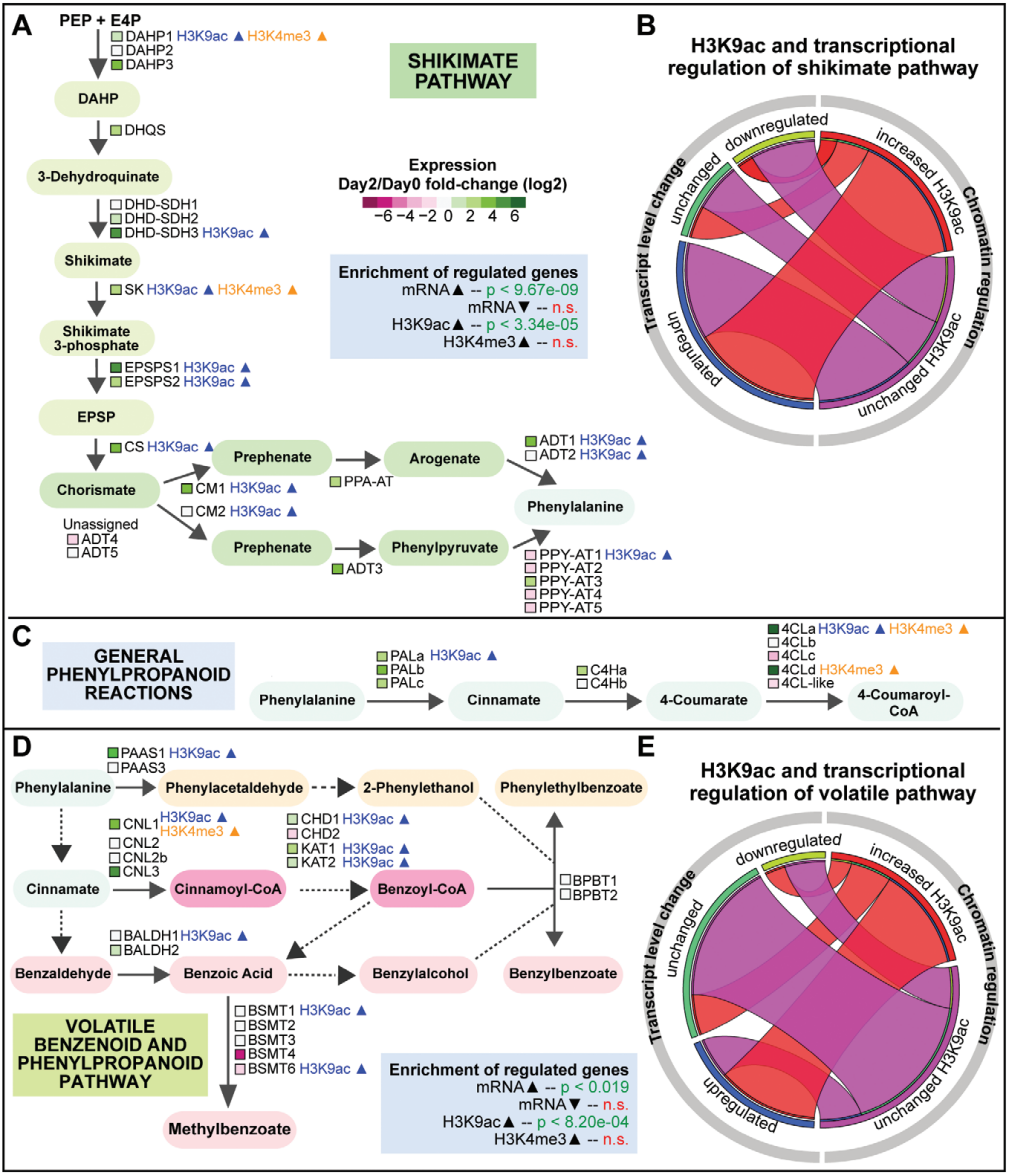
Transcriptional and epigenetic regulation of the shikimate, general phenylpropanoid, and volatile benzenoid/phenylpropanoid pathways. (A) Schematic of the shikimate and phenylalanine biosynthesis pathway with transcriptomic and epigenomic data overlaid. Color legend: shikimate pathway intermediates in light green, chorismate and phenylalanine synthesis intermediates in blue-green, and phenylalanine in light blue. The transcript level changes of genes underlying each enzymatic step are represented by the colored squares, with the color key shown in the graph (where green represents increased transcript levels and magenta represents decreased transcript levels). The significance of overlaps with DEGs and DMGs is determined by hypergeometric tests and is shown in the blue-shaded box. (B) Circos plot showing the two regulatory categories (transcriptional level changes and chromatin regulation) for genes involved in the shikimate pathway, as well as intercategorical relationships between the two regulatory categories. The plot consists of two semi-circles. The semi-circle on the left represents the transcriptional regulation categories for genes involved in the shikimate pathway: the number of genes that are down-regulated, up-regulated, or unchanged are proportional to the length of the three curved segments in dark blue, turquoise, and green, respectively. Similarly, the semi-circle on the right represents two chromatin regulatory categories for H3K9ac (increased or unchanged) for the same set of genes involved in the shikimate pathway. The genes shared between a transcriptional regulatory category (e.g. up-regulated) and a chromatin regulatory category (e.g. increased H3K9ac) are represented by the ribbon linking the two curved segments located within the two semi-circles. For example, the majority of the genes with increased H3K9ac are also up-regulated at the transcript level. (C) Schematic of the general phenylpropanoid genes generating shared metabolic intermediates (in light blue) with transcriptomic and epigenomic data overlaid. (D) Schematic of the volatile benzenoid and phenylpropanoid biosynthesis pathway with transcriptomic and epigenomic data overlaid for known genes. Color legend: general phenylpropanoid intermediates phenylalanine and cinnamate in light blue, phenylpropanoid-related (C_6_–C_2_) derived volatiles in orange, phenylpropanoid (C_6_–C_3_) and benzenoid (C_6_–C_1_) intermediates in magenta, and derived volatiles in pink. (E) Circos plot showing the two regulatory categories (transcriptional level changes and H3K9ac changes) and intercategorical relationships of volatile benzenoid and phenylpropanoid pathway genes. 4CL, 4-coumaryl-CoA ligase; ADT, arogenate dehydratase; BALDH, benzaldehyde dehydrogenase; BPBT, benzoyl-CoA:benzylalcohol/phenylethanol benzoyltransferase; BSMT, benzoic acid/salicylic acid carboxyl methyltransferase; C4H, cinnamate 4-hydroxylase; CHD, cinnamoyl-CoA hydratase-dehydrogenase; CM, chorismate mutase; CNL, cinnamoyl-CoA ligase; CS, chorismate synthase; DAHP, 3-deoxy-d-*arabino*-heptulosonate 7-phosphate; EPSPS, 5-*enol*pyruvylshikimate 3-phosphate; DHD-SDH, 3-dehydroquinate dehydratase and shikimate dehydrogenase; DHQS, 3-dehydroquinate synthase; KAT, 3-ketoacyl-CoA thiolase; PAAS, phenylacetaldehyde synthase; PAL, phenylalanine ammonia lyase; PPA-AT, prephenate aminotransferase; PPY-AT, phenylpyruvate aminotransferase; SK, shikimate kinase.

The enzymes involved in the shikimate and phenylalanine biosynthesis pathways have been well characterized in plants (Fig. 3A; Supplementary Table S5) (Maeda *et al.*, 2010, 2011; Maeda and Dudareva, 2012; Yoo *et al.*, 2013; Qian *et al.*, 2019). Overlaying the epigenomic and transcriptomic data revealed systematic H3K9ac deposition and transcriptional activation at almost every step of the pathway, for at least one gene copy if multiple genes are potentially involved in driving an enzymatic step (Fig. 3A). In total, 46% of the genes in this pathway display increased H3K9ac and, of those, 73% show increased gene expression (Fig. 3B). Statistically, genes encoding enzymes in this pathway are significantly enriched with DMGs gaining H3K9ac (overlap *P*-value <3.34E-05) and DEGs with increased transcript levels (overlap *P*-value <9.67E-09); in contrast, genes with a change in H3K4me3 levels do not appear significantly enriched. Our results therefore support that flux through the shikimate and phenylalanine biosynthetic pathways is a major target of H3K9ac and transcriptional regulation during petunia flower development to potentiate VOC production.

#### General phenylpropanoid reactions

From phenylalanine, a set of reactions catalyzed by phenylalanine ammonia lyase (PAL), cinnamate 4-hydroxylase (C4H), and 4-coumaryl-CoA ligase (4CL) generates metabolites shared by many downstream phenylpropanoid branches, including those toward synthesis of flavonoids, lignin, and VOCs (Ferrer *et al.*, 2008; Bombarely *et al.*, 2016) (Fig. 3C; Supplementary Table S5). In petunia, these enzymes are encoded by multiple gene loci (Bombarely *et al.*, 2016), with many showing increased transcript levels on day 2 relative to day 0 (Fig. 3C). H3K9ac deposition during anthesis is observed at one copy of *PAL* and one copy of *4CL*. In the case of *4CL*, two of the five copies are transcriptionally increased. Interestingly, the two induced copies also display increased H3K9ac or H3K4me3. 4CL drives the synthesis of 4-coumaryl-CoA, which has multiple fates in metabolism and can serve as a precursor for flavonoids and anthocyanins, lignin, and phenylpropene VOCs. The distinct chromatin and transcriptional regulation of these five copies could possibly indicate specialized gene functions for different metabolic fates.

#### Volatile benzenoid and phenylpropanoid pathways

The majority of volatile phenylpropanoid (C_6_–C_3_) and benzenoid (C_6_–C_1_) compounds are synthesized from cinnamate produced from phenylalanine by PAL, while formation of volatile phenylpropanoid-related (C_6_–C_2_) compounds occurs directly from phenylalanine (Dudareva *et al.*, 2013) (Fig. 3D). Genes encoding enzymes for this metabolic network (Supplementary Table S5) are significantly enriched with up-regulated DEGs and with DMGs having increased H3K9ac (Fig. 3D, E). Interestingly, increases in both H3K9ac levels and transcript levels occur at gene loci underlying the first few enzymatic steps in the pathway, notably phenylacetaldehyde synthase (PAAS), cinnamoyl-CoA ligase (CNL), cinnamoyl-CoA hydratase-dehydrogenase (CHD), and 3-ketoacyl-CoA thiolase (KAT). In contrast, the enzymes acting at the final steps of VOC formation producing methylbenzoate, benzylbenzoate, and phenylethylbenzoate are not induced at the transcript level (Fig. 3D). These end-step enzymes may rely on post-translational regulation or simply on increased precursor flux to generate the high levels of their products (Dudareva *et al.*, 2004; Clark *et al.*, 2009). Therefore, compared with the primary metabolism pathway (Fig. 3A) where nearly every enzymatic step is regulated at the chromatin level and transcript level, the regulation of the secondary metabolism pathway seems to show more target specificity, with a preference for enzymes of the early steps of the pathway.

#### Anthocyanin pathway

**Fig. 4.**
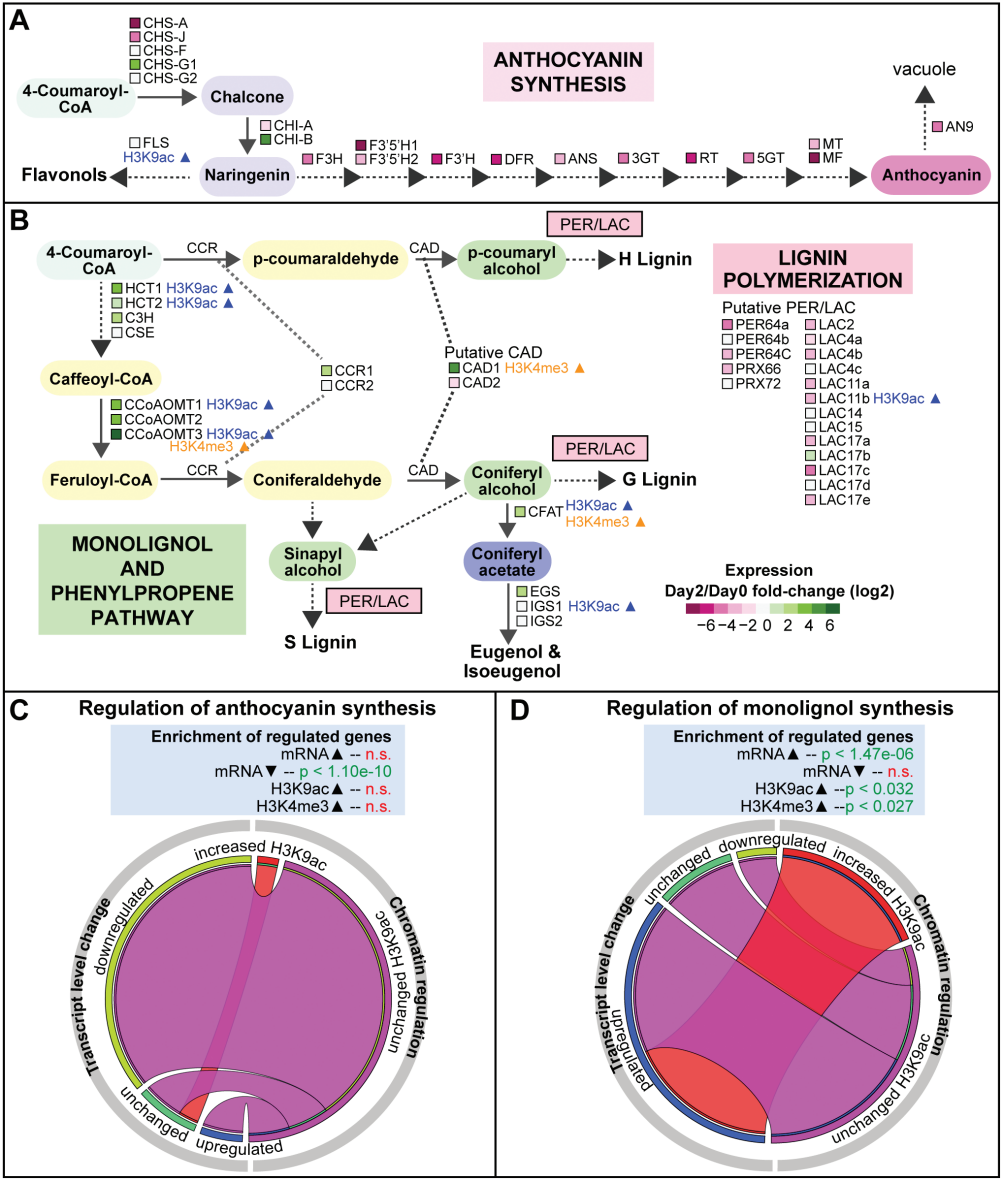
Transcriptional and epigenetic regulation of anthocyanin, lignin, and eugenol/isoeugenol biosynthesis. (A) Schematic of the flavonoid and anthocyanin biosynthesis pathway with transcriptomic data overlaid. Color legend: general phenylpropanoid intermediate 4-coumaroyl-CoA in light blue, flavonoid branch intermediates in dark blue, anthocyanin in magenta. (B) Schematic of the monolignol pathway genes generating the three monolignol precursors, the eugenol and isoeugenol synthesis branch, and the lignin polymerization peroxidase/laccase genes, with transcriptomic and epigenomic data overlaid. Color legend: general phenylpropanoid intermediate 4-coumaroyl-CoA in light blue, monolignol branch intermediates in yellow, monolignol units in green, phenylpropene branch intermediate coniferyl acetate in dark blue. (C) Regulatory categories of anthocyanin pathway genes and hypergeometric *P*-values determined for enrichment among DEGs and DMGs. (D) Regulatory categories of monolignol biosynthesis genes with hypergeometric *P*-values determined for enrichment among DEGs and DMGs. 3GT, 3-glucosyl transferase; 5GT, 5-glucosyl transferase; ANS, anthocyanin synthase; C3H, coumarate 3-hydroxylase; CAD, cinnamyl alcohol dehydrogenase; CCoAOMT, caffeoyl-CoA O-methyltransferase; CCR, cinnamoyl-CoA reductase; CFAT, coniferyl alcohol acetyltransferase; CHI, chalcone isomerase; CHS, chalcone synthase; CSE, caffeoyl shikimate esterase; DFR, dihydroflavonol 4-reductase; EGS, eugenol synthase; F3′5′H, flavonoid 3′,5′-hydroxylase; F3H, flavanone 3-hydroxylase; F3′H, flavonoid 3′-hydroxylase; FLS, flavonol synthase; HCT, hydroxycinnamoyl-CoA:shikimate/quinate hydroxycinnamoyl transferase; IGS, isoeugenol synthase; LAC, laccase; MF, methylation at five; MT, methylation at three; PER/PRX, peroxidase; RT, rhamnosylation at three.

Certain floral VOCs such as eugenol and isoeugenol are synthesized from 4-coumaroyl-CoA, which is also utilized to produce the pigment anthocyanin (Quattrocchio *et al.*, 1999). In *P. hybrida* cv. Mitchell, the flowers emit high levels of VOCs but the petals lack pigmentation. Our examination of the anthocyanin biosynthesis pathway (Fig. 4A; Supplementary Table S6) (Quattrocchio *et al.*, 1993; Ferrer *et al.*, 2008; Bombarely *et al.*, 2016; Passeri *et al.*, 2016) found that transcriptional repression shuts off the anthocyanin branch post-anthesis, possibly contributing to the channeling of carbon flux toward VOC production (Fig. 4A). Indeed, anthocyanin pathway genes are significantly enriched with down-regulated DEGs (Fig. 4C). At the chromatin level, increased H3K9ac deposition during corolla development is almost completely excluded from the anthocyanin synthesis pathway. Overall, these results demonstrate that during the maturation of petunia corolla, there is a corresponding deactivation of genes involved in anthocyanin production concurrent with the activation of VOC branches of the phenylpropanoid pathway.

#### Monolignol/phenylpropene synthesis pathway

Our analysis revealed a complex and intriguing regulation pattern of the lignin biosynthetic branch of the phenylpropanoid pathway, with significant enrichment of lignin biosynthesis genes among both up-regulated and down-regulated genes on day 2 versus day 0 (Fig. 2E). It should be noted that volatile phenylpropene and lignin biosynthesis share the biochemical steps up to monolignol biosynthesis, where they diverge. As such, further investigation of the pathway revealed important regulatory control points to direct flux through the monolignol pathway to promote production of volatile eugenol and isoeugenol, and repress synthesis of lignin polymers in the mature corolla.

In the monolignol biosynthetic pathway (Supplementary Table S7), enzymes including hydroxycinnamoyl-CoA:shikimate/quinate hydroxycinnamoyl transferase (HCT), caffeoyl-CoA *O*-methyltransferase (CCoAOMT), and coumarate 3-hydroxylase (C3H) utilize 4-coumaroyl-CoA from the general phenylpropanoid pathway to produce aldehyde intermediates *p*-coumaraldehyde or coniferaldehyde (Fig. 4B) (Muhlemann *et al.*, 2014*b*; Shaipulah *et al.*, 2016; Kim *et al.*, 2019). CAD enzyme(s) then convert the aldehyde intermediates into three monolignol intermediates, *p*-coumaryl alcohol, sinapyl alcohol, and coniferyl alcohol. Genes encoding these enzymes are induced on day 2 relative to day 0 (Fig. 4B). Specifically, genes encoding the enzymes catalyzing the early steps (*HCT* and *CCoAOMT*) are targeted for H3K9ac deposition during corolla maturation (Fig. 4B). Overall, there is a significant enrichment of up-regulated DEGs and DMGs with increased H3K9ac among the genes contributing to the production of the monolignol intermediates (Fig. 4D), indicating that chromatin and transcriptional regulation underlie the increased flux through the monolignol pathway.

Two competing fates exist for these monolignol intermediates in petunia flowers. The monolignol can be used to synthesize lignin polymers for incorporation into the cell wall through the function of specialized peroxidases (PERs) and laccases (LACs) (Fraser and Chapple, 2011) (Fig. 4B). Alternatively, the monolignol coniferyl alcohol can serve as a precursor for production of the phenylpropene VOCs eugenol and isoeugenol (Muhlemann *et al.*, 2014*b*), through the actions of coniferyl alcohol acetyltransferase (CFAT) (Dexter *et al.*, 2007) and eugenol synthase (EGS) or isoeugenol synthase (IGS) (Koeduka *et al.*, 2008) (Fig. 4B). Both *CFAT* and *EGS* are induced in the corolla at the transcript level, while *CFAT* and *IGS1* show increased H3K9ac levels. In contrast, we observed a clear pattern of decreased transcript levels for *PER* and *LAC* (Fig. 4B; Supplementary Table S7), which is in agreement with low levels of lignification observed in petunia flower relative to stem and leaf tissue (Muhlemann *et al.*, 2014*b*; Shaipulah *et al.*, 2016).

Overall, our results suggest that important control points of the monolignol/phenylpropene metabolic network are regulated at the chromatin level, possibly to direct flux through the monolignol pathway to produce VOCs. The apparent GO term enrichment for lignin biosynthesis in both up- and down-regulated genes (Fig. 2E) is due to the separate regulatory regimes governing the different modules of the lignin branch of phenylpropanoid metabolism; production of monolignol intermediates is activated to produce phenylpropene VOCs in petunia, while at the same time there is a decreased incorporation of monolignol subunits into lignin polymers.

### Histone acetylation is essential for transcriptional activation of VOC genes and VOC emission

**Fig. 5.**
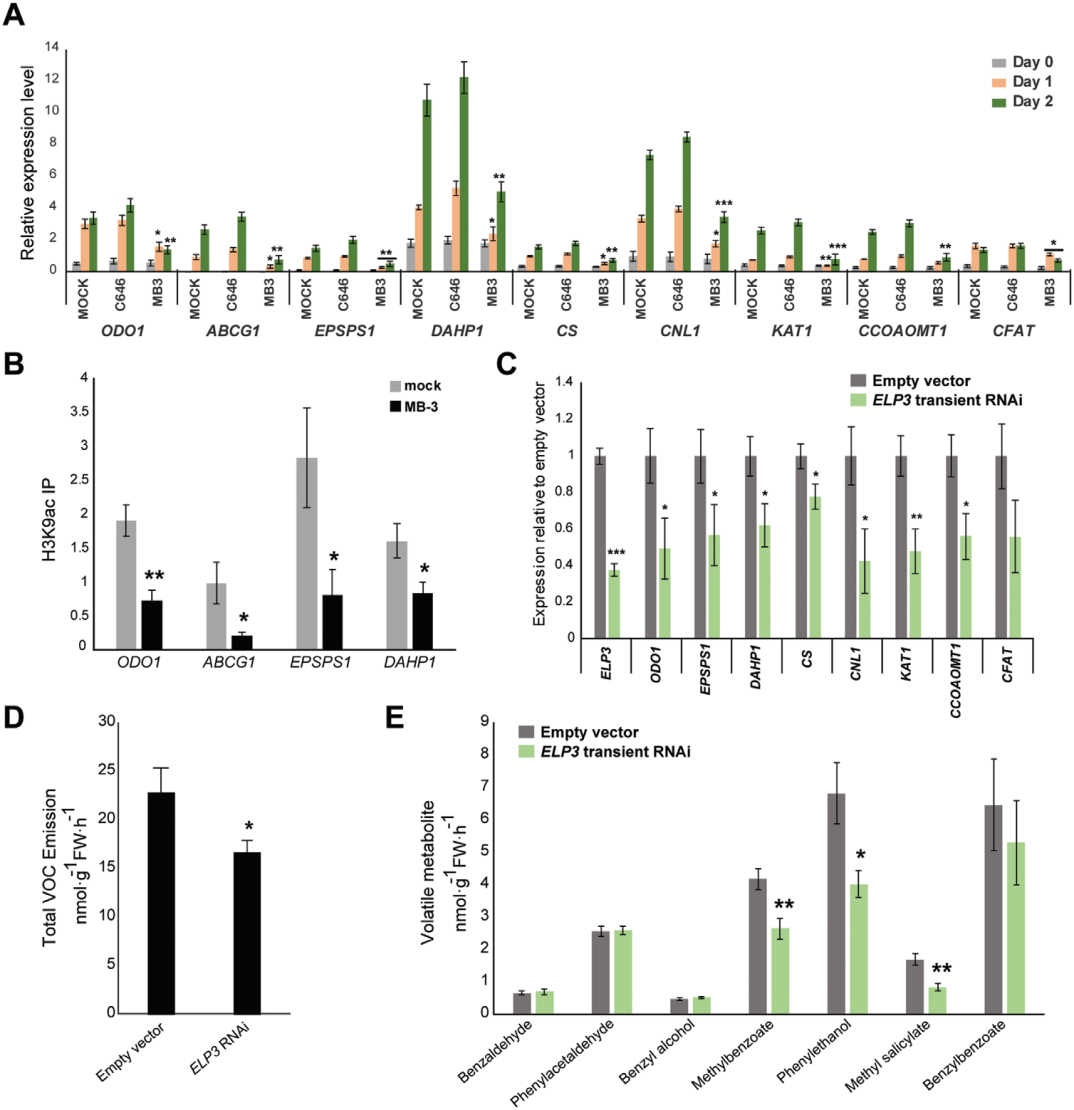
Inhibition of histone acetylation affects VOC gene transcription and VOC emission. (A) qRT–PCR results showing that the induction of VOC genes is impaired by the HAT inhibitor MB-3, but not by C646. The relative expression levels normalized to reference genes *UBQ10* and *FBP1* are shown. Error bars represent the SEM for biological replicates (*n*=3–4). (B) ChIP-qPCR results showing a significant reduction of H3K9ac in the MB-3-treated samples compared with the mock samples. Detached flower buds at day 0 were treated with mock (0.1% DMSO) or MB-3 (100 μM in 0.1% DMSO) for 2 d and then processed for ChIP with antibodies against H3K9ac. qPCR was performed with primers designed for the gene body around the acetylation peaks based on the ChIP-Seq data in this study. Immunoprecipitation (IP) of ChIP samples was first normalized to the input DNA and then normalized to housekeeping genes *ACTIN* and *PP2AA3*. Significance of difference between mock samples and MB-3-treated samples was determined by Student’s *t*-test (*n*=4) and shown as asterisks: *P<0.05, **P<0.01. (C) qRT–PCR results showing that transient RNAi successfully suppresses the expression of *ELP3* as well as reducing the expression level of VOC genes. Six biological replicates of independent transformation events were included. The relative expression levels were determined by qRT–PCR, normalized to *UBQ10* and *FBP1*, and scaled relative to empty vector control. Error bars represent the SEM (*n*=6). (D and E) Floral VOC emission from petunia flowers measured as total emission (D) or individual metabolites (E) in *ELP3* transient RNAi samples and empty vector control. Significance of difference between treatment and mock (A) or between *ELP3* RNAi and the empty vector (C–E) is determined by Student’s *t*-test and shown by asterisks: *P≤0.05, **P≤0.01, ***P≤0.001. Error bars represent the SEM for biological replicates from independent transformations (*n*=6).

To determine whether the observed dynamic histone acetylation has a causal effect on the induction of VOC genes, we treated petunia flower buds with chemical inhibitors of HATs and tested whether the activation of VOC genes during anthesis was impeded. In detail, petunia buds on day 0 were treated with C646 or MB-3, which are small molecule inhibitors of HATs (Malapeira *et al.*, 2012; Lee *et al.*, 2015; Aquea *et al.*, 2017). qRT–PCR was then performed to measure the gene expression on days 0, 1, and 2 for a group of genes with increased H3K9ac and transcriptional activation during anthesis in our ChIP-Seq and RNA-Seq analysis (Supplementary Datasets S1, S2), selected from different branches of VOC pathways: phenylalanine biosynthesis (*EPSPS1*, *DAHP1*, and *CS*), benzenoid VOC biosynthesis (*CNL1* and *KAT1*), and phenylpropene VOC biosynthesis (*CCoAOMT1* and *CFAT*). Also included were *ODORANT1* (*ODO1*), encoding a transcription factor involved in activating shikimate pathway and VOC genes (Verdonk *et al.*, 2005), and *ATP-binding cassette subfamily G member 1* (*PhABCG1*), encoding an active VOC transporter required for VOC emission (Adebesin *et al.*, 2017). The tested genes showed activation of gene expression during anthesis in mock-treated samples and, strikingly, all the genes exhibited a notable attenuation of transcriptional activation when treated with MB-3 (Fig. 5A). The histone acetylation levels at select gene loci were measured on day 2 using ChIP-qPCR, and H3K9ac showed significant reduction in the MB-3-treated samples compared with the mock-treated samples, supporting that the attenuation of transcriptional activation was indeed due to reduced HAT activity (Fig. 5B). H3K14ac levels were unaffected (Supplementary Fig. S4B), suggesting that MB-3 particularly affects the H3K9ac levels at this developmental stage. Together, these results support the mechanistic importance of H3K9ac deposition in activating primary and secondary metabolic pathways for VOC synthesis and emission in the petunia corolla. Treatment with the other inhibitor, C646, did not alter the transcriptional activation of any tested genes (Fig. 5A). MB-3 inhibits the GNAT family HATs and to a lesser extent p300/CBP family HATs, while C646 mainly inhibits the p300/CBP family (Biel *et al.*, 2004; Bowers *et al.*, 2010). Our results thus suggest that unknown member(s) of the GNAT family probably have a specialized role in the transcriptional regulation of VOC biosynthesis.

To determine which member of the GNAT family is responsible for the deposition of H3K9ac that influences VOC activation, the HAT family genes were identified in the *P. axillaris* genome based on homology with Arabidopsis HAT genes (Pandey *et al.*, 2002) (Supplementary Table S8). Among the identified HAT genes, *ELP3*, a GNAT family HAT, was unique in being significantly up-regulated (FDR <0.05; fold change >2) during corolla development (Supplementary Dataset S2; Supplementary Fig. S5A). We thus hypothesized that ELP3 mediates VOC gene expression in petunia flower during anthesis. To test this hypothesis, petunia *ELP3* was down-regulated in flowers using transient RNAi strategy. *ELP3* RNAi was performed in six biological replicates, each representing independent transformation events, and the expression of select VOC genes and levels of emitted VOCs were measured 2 d post-infiltration. We observed an average reduction by 62% in *ELP3* transcript levels (Fig. 5C), with only minor changes in expression levels of other HAT genes (Supplementary Fig. S5B). Knockdown of *ELP3* resulted in significant down-regulation of most VOC biosynthetic genes assayed (Fig. 5C), suggesting that the expression of these VOC genes is dependent on ELP3. Importantly, the total emitted volatiles were also decreased in *ELP3* RNAi flowers relative to empty vector control (Fig. 5D), with individual compounds affected to different degrees (Fig. 5E). Taken together, these RNAi results along with chemical inhibition experiments provide two lines of *in planta* evidence that histone acetylation, mediated at least in part by ELP3, activates transcription of VOC metabolic pathways, which is required for the synthesis and emission of volatile benzenoid products.

### Epigenetic and transcriptional activation of hormone signaling pathways during anthesis

**Fig. 6.**
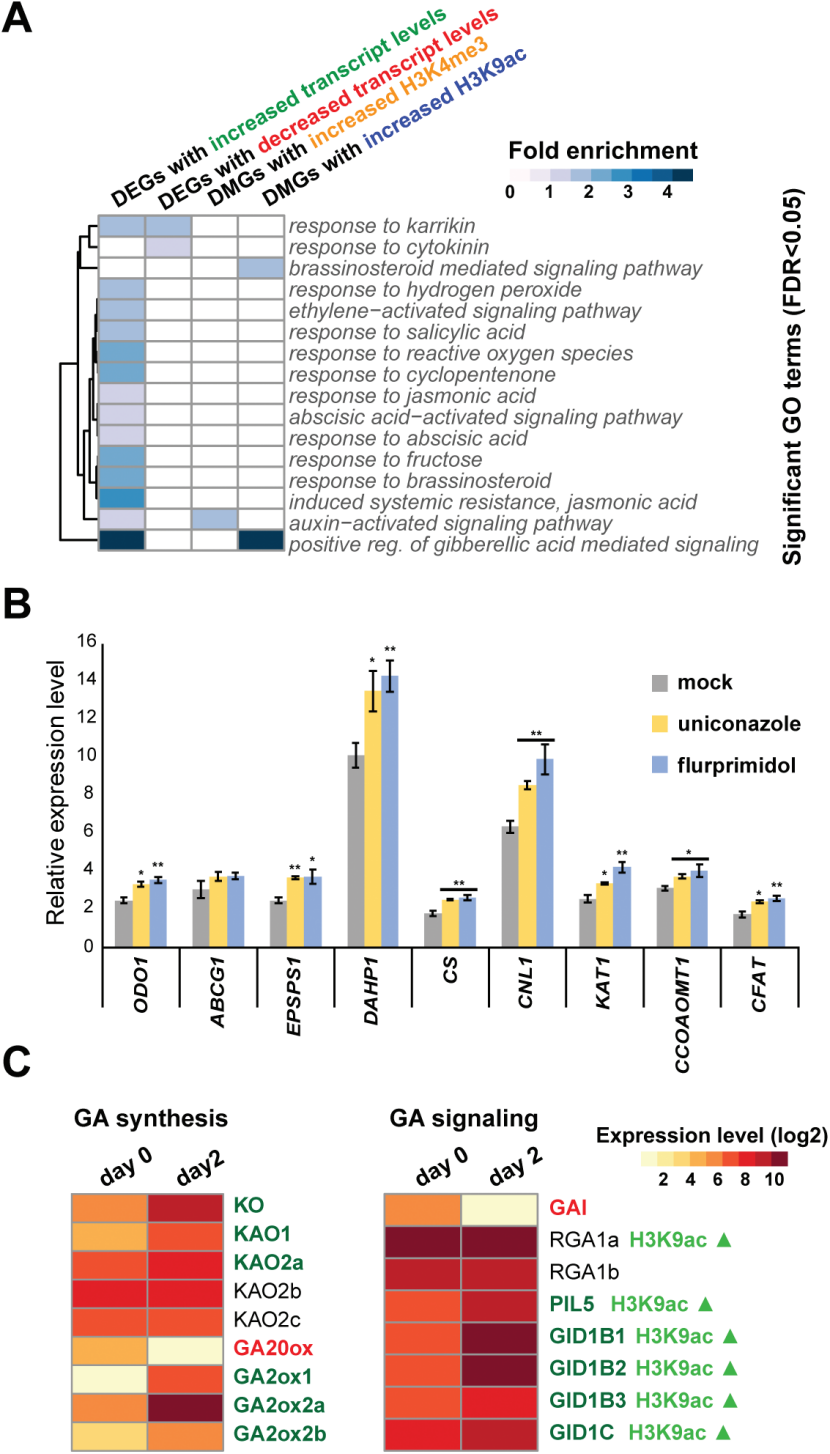
Phytohormone and gibberellic acid (GA) signaling in petunia corolla. (A) Heatmap depicting the fold enrichment of signaling GO terms significantly enriched among the DEGs or DMGs. (B) qRT–PCR results showing the up-regulation of VOC genes when GA synthesis is inhibited by uniconazole or flurprimidol. Relative expression levels normalized to *UBQ10* and *FBP1* are shown, and error bars represent the SEM for biological replicates (*n*=3–4). Significance of treatment relative to mock is determined by Student’s *t*-test and shown as asterisks: *P≤0.05, **P≤0.01, ***P≤0.001. (C) Heatmap depicting expression levels of GA synthesis and signaling genes determined by RNA-seq. Up-regulated DEGs are shown in green, and down-regulated DEGs are shown in red. Log2-normalized expression levels are shown for genes whose expression levels are above the first quartile of all genes in the corolla at either day 0 or day 2. Genes names reflect best homology to Arabidopsis gene copies, and multiple expressed homologs were named alphabetically a–c or, for *GID1B*, copies 1–3. GAI, GA-insensitive; GID, GA-insensitive dwarf; KO, *ent*-kaurene oxidase; KAO, *ent*-kaurene acid oxidase; PIL, phytochrome-interacting factor 3-like; RGA, repressor of ga1-3.

To further investigate the regulatory and signaling mechanisms that govern VOC production and release, we examined GO terms involved in plant signaling for their significant enrichment among the DMGs and DEGs (Fig. 6A). Interestingly, ‘positive regulation of gibberellic acid (GA) mediated signaling pathway’ was the only significantly enriched GO term shared between transcriptionally activated DEGs and DMGs gaining H3K9ac.

It has been reported that GA represses VOC synthesis and emission (Ravid *et al.*, 2017). Thus, it would be reasonable to expect that GA biosynthesis and signaling are down-regulated when VOC emission is induced. In contrast, our results revealed up-regulation of GA signaling genes during the developmental time frame of VOC induction (Fig. 6A). To investigate this discrepancy, we first tested whether GA serves as a positive or negative regulator of VOC genes in our system. Flower buds were treated with 100 µM uniconazole or flurprimidol, inhibitors of GA synthesis, or mock control (Yamaguchi *et al.*, 2001; Pullman *et al.*, 2005), and the expression levels of selected VOC genes on day 2 were analyzed by qRT–PCR. Inhibition of GA biosynthesis led to a moderate but statistically significant increase in the transcript levels of most VOC genes assayed (Fig. 6B). Therefore, our results agree with the previous report (Ravid *et al.*, 2017) that GA represses VOC synthesis. Next, we took advantage of our genome-wide data to examine the temporal regulation of GA synthesis and GA signaling pathways during anthesis (Fig. 6C; Supplementary Table S9). Interestingly, GA biosynthesis and GA signaling genes show distinct regulation patterns. The GA biosynthetic genes show mixed expression patterns and lack dynamic histone acetylation (Fig. 6C). Notably, although *ent-KAURENE OXIDASE* (*KO*) and *ent-KAURENE ACID OXIDASE* (*KAO*) are well expressed, *GA20ox*, which acts downstream of KAO to produce bioactive GA, is strongly down-regulated from day 0 to day 2 (Fig. 6C). Meanwhile, several copies of *GA2ox*, which convert bioactive GA to inactive forms, are significantly and strongly induced from day 0 to day 2 (Fig. 6C). Our data thus suggest that the biosynthesis of GA in the corolla is down-regulated post-anthesis. In contrast, many genes involved in GA perception and signaling are up-regulated with increased H3K9ac during anthesis, notably including four copies of GA receptor *GIBBERELLIN INSENSITIVE DWARF1* (*GID1*) (Tyler *et al.*, 2004; Murase *et al.*, 2008) and one copy of *REPRESSOR OF GA 1–3* (*RGA1*) and *PHYTOCHROME INTERACTING FACTOR3-LIKE 5* (*PIL5*), a positive regulator of *RGA1* (Oh *et al.*, 2007). Overall, our integrated ChIP-Seq and RNA-Seq analyses uncovered an intriguing role for GA signaling in regulating VOC production, suggesting low GA synthesis but high GA sensitivity during peak VOC emission. It is possible that GA may repress the VOC pathway before the flower opens, and the reduction in GA levels after flower opening allows high levels of VOC emission. Meanwhile, the corolla tissues become highly sensitive to GA, as the GA level may rise again during senescence or after pollination to shut off the VOC emission.

## Discussion

**Fig. 7.**
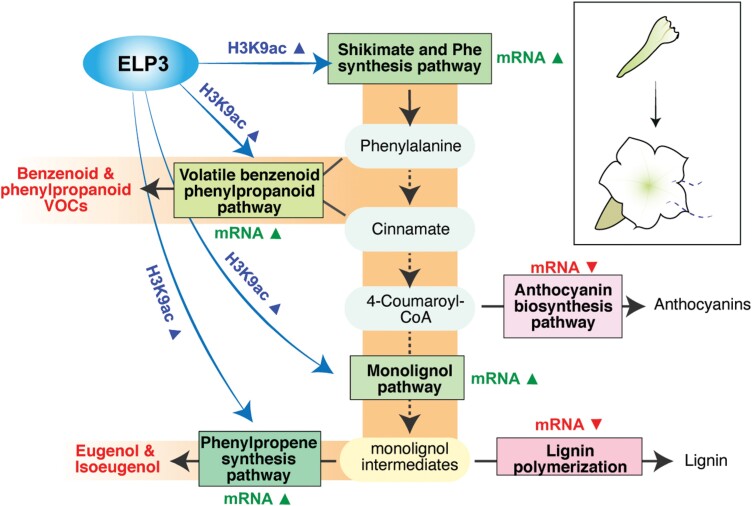
Summary model of chromatin-level regulation and transcriptional reprogramming to facilitate VOC emission during petunia corolla development. This schematic summarizes regulation of phenylalanine and phenylpropanoid synthesis branches observed at the chromatin level through increased histone acetylation (H3K9ac) at day 2 post-anthesis compared with day 0 bud tissue in the petunia corolla, and corresponding transcriptional changes in order to direct metabolic flow through the phenylpropanoid pathway to generate floral VOCs.

Rapid and efficient production of secondary metabolites in response to developmental and environmental stimuli requires coordinated regulation of primary and secondary metabolic pathways. By integrating ChIP-Seq and RNA-Seq analyses, we have shown that chromatin-level regulation acts as an underlying mechanism activating primary and secondary metabolic networks leading to formation of floral VOCs during flower development in petunia. Our data revealed an exquisite coordination balancing substrate availability and flux via different metabolite fates through dynamic regulation of chromatin modification and transcript levels of metabolic gene networks (Fig. 7). While chromatin modification and transcriptional reprogramming ensure that both large amounts of phenylalanine substrate are available and it flows preferentially to VOC biosynthesis, other metabolic fates are blocked by transcriptional inactivation of those branches. In detail, during flower opening and post-anthesis, H3K9ac is deposited at gene loci encoding enzymes involved in the shikimate and phenylalanine biosynthesis pathways, with a corresponding increase in transcriptional activity, enabling the production of large amounts of phenylalanine (Figs 3A, 7). Phenylalanine is in turn a precursor for the volatile benzenoid and phenylpropanoid network, in which the enzymes involved are regulated in a targeted manner through increased H3K9ac and up-regulated transcript levels to direct metabolic flux to VOC production (Figs 3D, 7). Phenylalanine is also converted to 4-coumaroyl-CoA, which is a common substrate of divergent phenylpropanoid branches including flavonoid/anthocyanin synthesis and monolignol biosynthesis. Targeted H3K9ac and transcript level increases are observed in the monolignol synthesis pathway and the enzymes that produce the phenylpropene VOCs eugenol and isoeugenol (Figs 4B, 7), while there is a simultaneous deactivation of lignin polymerization (Figs 4B, 7) and anthocyanin biosynthesis (Figs 4A, 7), with the net effect of directing 4-coumaroyl-CoA substrate toward phenylpropene VOC production.

Our findings in petunia share similarity with the epigenetic control of secondary metabolism in fungi, where HATs contribute to the transcriptional activation of biosynthetic gene clusters and production of a range of specific secondary metabolites (Strauss and Reyes-Dominguez, 2011; Pfannenstiel and Keller, 2019). Our data support that H3K9ac is a specific histone mark contributing toward activating the VOC synthesis pathway, rather than merely a permissive histone mark associated with activated genes. Importantly, blocking HAT activity by chemical inhibition attenuates the normal transcriptional induction of these pathways, thus supporting that *de novo* histone acetylation is required for the VOC synthesis (Fig. 5). Indeed, another common permissive histone mark assayed in our study, H3K4me3, was not significantly associated with VOC production during anthesis. One possible explanation for this specific role of histone acetylation is that among the different histone modifications, histone acetylation is hypothesized to integrate information on the cellular metabolic status into chromatin states because it uses acetyl-CoA as substrate (Dutta *et al.*, 2016; Reid *et al.*, 2017).

Histone acetylation is ‘written’ by HAT family proteins. We found that a specific HAT, ELP3, activates multiple genes in the VOC gene network (Fig. 7), by showing that transient RNAi knockdown of *ELP3* attenuates the transcription of genes in the VOC pathways as well as the level of emitted volatile metabolites (Fig. 5C–E). ELP3, short for Elongator complex subunit 3, was first discovered through association with RNA polymerase II in yeast (Otero *et al.*, 1999; Krogan and Greenblatt, 2001), and later was found to be conserved among archaea, bacteria, yeast, animals, and plants. The elongator complex consists of six subunits, of which ELP3 is the catalytic subunit with HAT activity. The *elp3* mutants in plants have narrow leaves (Nelissen *et al.*, 2005, 2010) and altered root morphology (Jia *et al.*, 2015; Qi *et al.*, 2019). Previous studies suggest a role for ELP3 in regulating gene transcription with target specificity primarily in dividing meristemic tissues (Nelissen *et al.*, 2010; Jia *et al.*, 2015; Qi *et al.*, 2019). Our findings now link ELP3 with activation of the VOC metabolic network in mature flowers, where it is likely to be responsible for deposition of H3K9ac along the gene body of VOC genes to facilitate high levels of transcription.

The mechanistic insights obtained in this study on petunia corolla provide a framework to understand epigenetic regulatory mechanisms underlying plant secondary metabolic pathways, the activities of which are often restricted by developmental and environmental contexts. Importantly, when VOC pathways are activated in the corolla, coordinated H3K9ac deposition and transcriptional activation mainly occur at gene loci encoding enzymes critical for directing flow of shared precursor metabolites toward VOC formation. This is the case for *DAHP1* in the shikimate pathway (Fig. 3A), *CM1* in phenylalanine biosynthesis (Fig. 3A), *PAAS1* and *CNL1* in volatile benzenoid and phenylpropanoid biosynthesis (Fig. 3D), *HCT1* and *HCT2* in monolignol biosynthesis (Fig. 4B), and *CFAT* in eugenol and isoeugenol biosynthesis (Fig. 4B). How such target specificity is achieved, from biochemical to evolutionary scales, is an intriguing question that requires further investigation.

Finally, the uncovered chromatin-level regulation of secondary metabolic pathways has practical implications in metabolic engineering. Engineering of phenylpropanoid and VOC metabolism has been a topic of interest (Dudareva and Negre, 2005; Brilli *et al.*, 2019; Plasmeijer *et al.*, 2020), as VOCs provide economically important flavor and scent characteristics to food and botanical products (Dudareva *et al.*, 2013). It was noted that an explicit control of metabolic flow through targeted primary and secondary metabolic pathways is required in order to produce high levels of desired specialized metabolites. Our results showed that these metabolic networks are regulated at chromatin level through histone modifications such as H3K9ac. Therefore, the optimal outcomes of metabolic engineering will require a mindset toward network-level regulation of primary and secondary metabolism, and efforts may be critically impacted by the local chromatin environment of transgenes.

## Supplementary data

The following supplementary data are available at *JXB* online.

Fig. S1. ChIP-Seq read alignment to petunia genomes.

Fig. S2. RNA-Seq read alignment to petunia genomes.

Fig. S3. Decreased H3K9ac DMGs.

Fig. S4. ChIP-qPCR of MB-3-treated petunia.

Fig. S5. Expression of histone acetyltransferases (HATs) in petunia.

Dataset S1. Increased and decreased H3K9ac and H3K4me3 DMGs.

Dataset S2. Transcriptionally activated and repressed DEGs.

Table S1. Primers used in the study.

Table S2. Enriched GO terms among DMGs with increased H3K9ac.

Table S3. Enriched GO terms among DMGs with increased H3K4me3.

Table S4. Enriched GO terms among up-regulated (A) or down-regulated (B) DEGs.

Table S5. Genes involved in shikimate and phenylalanine synthesis, the general phenylpropanoid pathway, or volatile benzenoid and phenylpropanoid synthesis genes.

Table S6. Flavonoid and anthocyanin synthesis genes.

Table S7. Genes involved in the monolignol pathway, eugenol/isoeugenol synthesis, or lignin polymerization.

Table S8. Histone acetyltransferase genes.

Table S9. Gibberellic acid signaling and metabolism genes.

erab072_suppl_Supplementary-File-1

erab072_suppl_Supplementary-File-2

erab072_suppl_Supplementary-File-3

## Data Availability

ChIP-Seq data generated in this work are available at the NCBI Sequence Read Archive under BioProject accession PRJNA650505.

## Abbreviations

ChIP-Seq: ChIP squencing
DEG: differentially expressed gene
DMG: differentially modified gene
ELP3: elongator complex subunit 3
FDR: false discovery rate
GNAT/MYST: Gcn5-related *N*-acetyltransferase/MOZ, Ybf2/Sas3, Sas2, Tip60 family
GO: Gene Ontology
HAT: histone acetyltransferase
H3K9ac: histone subunit 3 lysine 9 acetylation
H3K4me3: histone subunit 3 lysine 4 trimethylation
VOC: volatile organic compound

## References

[CIT0001] Adebesin F , Widhalm JR, Boachon B, et al. 2017. Emission of volatile organic compounds from petunia flowers is facilitated by an ABC transporter. Science 356, 1386–1388. 28663500 10.1126/science.aan0826

[CIT0002] Affek HP , Yakir D. 2002. Protection by isoprene against singlet oxygen in leaves. Plant Physiology 129, 269–277. 12011357 10.1104/pp.010909 PMC155890

[CIT0003] Ameye M , Audenaert K, De Zutter N, Steppe K, Van Meulebroek L, Vanhaecke L, De Vleesschauwer D, Haesaert G, Smagghe G. 2015. Priming of wheat with the green leaf volatile Z-3-hexenyl acetate enhances defense against *Fusarium graminearum* but boosts deoxynivalenol production. Plant Physiology 167, 1671–1684. 25713338 10.1104/pp.15.00107 PMC4378182

[CIT0004] Anders S , Pyl PT, Huber W. 2015. HTSeq—a Python framework to work with high-throughput sequencing data. Bioinformatics 31, 166–169. 25260700 10.1093/bioinformatics/btu638 PMC4287950

[CIT0005] Aquea F , Timmermann T, Herrera-Vásquez A. 2017. Chemical inhibition of the histone acetyltransferase activity in *Arabidopsis thaliana*. Biochemical and Biophysical Research Communications 483, 664–668. 27993678 10.1016/j.bbrc.2016.12.086

[CIT0006] Arimura G , Huber DP, Bohlmann J. 2004. Forest tent caterpillars (*Malacosoma disstria*) induce local and systemic diurnal emissions of terpenoid volatiles in hybrid poplar (*Populus trichocarpa* × *deltoides*): cDNA cloning, functional characterization, and patterns of gene expression of (–)-germacrene D synthase, PtdTPS1. The Plant Journal 37, 603–616. 14756770 10.1111/j.1365-313x.2003.01987.x

[CIT0007] Benjamini Y , Hochberg Y. 1995. Controlling the false discovery rate: a practical and powerful approach to multiple testing. Journal of the Royal Statistical Society: Series B (Methodological) 57, 289–300.

[CIT0008] Berardini TZ , Mundodi S, Reiser L, et al. 2004. Functional annotation of the Arabidopsis genome using controlled vocabularies. Plant Physiology 135, 745–755. 15173566 10.1104/pp.104.040071 PMC514112

[CIT0009] Biel M , Kretsovali A, Karatzali E, Papamatheakis J, Giannis A. 2004. Design, synthesis, and biological evaluation of a small-molecule inhibitor of the histone acetyltransferase Gcn5. Angewandte Chemie (International Edition) 43, 3974–3976. 15274229 10.1002/anie.200453879

[CIT0010] Boachon B , Lynch JH, Ray S, Yuan J, Caldo KMP, Junker RR, Kessler SA, Morgan JA, Dudareva N. 2019. Natural fumigation as a mechanism for volatile transport between flower organs. Nature Chemical Biology 15, 583–588. 31101916 10.1038/s41589-019-0287-5

[CIT0011] Bombarely A , Moser M, Amrad A, et al. 2016. Insight into the evolution of the Solanaceae from the parental genomes of *Petunia hybrida*. Nature Plants 2, 16074. 27255838 10.1038/nplants.2016.74

[CIT0012] Borges RM , Bessière JM, Hossaert-McKey M. 2008. The chemical ecology of seed dispersal in monoecious and dioecious figs. Functional Ecology 22, 484–493.

[CIT0013] Bowers EM , Yan G, Mukherjee C, et al. 2010. Virtual ligand screening of the p300/CBP histone acetyltransferase: identification of a selective small molecule inhibitor. Chemistry & Biology 17, 471–482. 20534345 10.1016/j.chembiol.2010.03.006 PMC2884008

[CIT0014] Brilli F , Loreto F, Baccelli I. 2019. Exploiting plant volatile organic compounds (VOCs) in agriculture to improve sustainable defense strategies and productivity of crops. Frontiers in Plant Science 10, 264. 30941152 10.3389/fpls.2019.00264 PMC6434774

[CIT0015] Brodmann J , Twele R, Francke W, Yi-bo L, Xi-qiang S, Ayasse M. 2009. Orchid mimics honey bee alarm pheromone in order to attract hornets for pollination. Current Biology 19, 1368–1372. 19664924 10.1016/j.cub.2009.06.067

[CIT0016] Byers KJ , Bradshaw HD Jr, Riffell JA. 2014. Three floral volatiles contribute to differential pollinator attraction in monkeyflowers (*Mimulus*). Journal of Experimental Biology 217, 614–623. 10.1242/jeb.092213 PMC3922836 24198269

[CIT0017] Camacho C , Coulouris G, Avagyan V, Ma N, Papadopoulos J, Bealer K, Madden TL. 2009. BLAST+: architecture and applications. BMC Bioinformatics 10, 421. 20003500 10.1186/1471-2105-10-421 PMC2803857

[CIT0018] Cheng CY , Krishnakumar V, Chan AP, Thibaud-Nissen F, Schobel S, Town CD. 2017. Araport11: a complete reannotation of the *Arabidopsis thaliana* reference genome. The Plant Journal 89, 789–804. 27862469 10.1111/tpj.13415

[CIT0019] Chinnusamy V , Zhu JK. 2009. Epigenetic regulation of stress responses in plants. Current Opinion in Plant Biology 12, 133–139. 19179104 10.1016/j.pbi.2008.12.006 PMC3139470

[CIT0020] Clark DG , Pichersky E, Verdonk J, Dudareva N, Haring M, Klahre U, Schuurink R. 2009. Benzenoids dominate the fragrance of petunia flowers. In: Gerats T, Strommer J, eds. Petunia: evolutionary, developmental and physiological genetics. New York: Springer, 51–69.

[CIT0021] Colquhoun TA , Verdonk JC, Schimmel BC, Tieman DM, Underwood BA, Clark DG. 2010. Petunia floral volatile benzenoid/phenylpropanoid genes are regulated in a similar manner. Phytochemistry 71, 158–167. 19889429 10.1016/j.phytochem.2009.09.036

[CIT0022] De Moraes CM , Mescher MC, Tumlinson JH. 2001. Caterpillar-induced nocturnal plant volatiles repel conspecific females. Nature 410, 577–580. 11279494 10.1038/35069058

[CIT0023] Dexter R , Qualley A, Kish CM, Ma CJ, Koeduka T, Nagegowda DA, Dudareva N, Pichersky E, Clark D. 2007. Characterization of a petunia acetyltransferase involved in the biosynthesis of the floral volatile isoeugenol. The Plant Journal 49, 265–275. 17241449 10.1111/j.1365-313X.2006.02954.x

[CIT0024] Du Z , Li H, Wei Q, et al. 2013. Genome-wide analysis of histone modifications: H3K4me2, H3K4me3, H3K9ac, and H3K27ac in *Oryza sativa* L. *japonica*. Molecular Plant 6, 1463–1472. 23355544 10.1093/mp/sst018 PMC3842134

[CIT0025] Dudareva N , Cseke L, Blanc VM, Pichersky E. 1996. Evolution of floral scent in *Clarkia*: novel patterns of S-linalool synthase gene expression in the *C. breweri* flower. The Plant Cell 8, 1137–1148. 8768373 10.1105/tpc.8.7.1137 PMC161191

[CIT0026] Dudareva N , Klempien A, Muhlemann JK, Kaplan I. 2013. Biosynthesis, function and metabolic engineering of plant volatile organic compounds. New Phytologist 198, 16–32. 10.1111/nph.12145 23383981

[CIT0027] Dudareva N , Martin D, Kish CM, Kolosova N, Gorenstein N, Fäldt J, Miller B, Bohlmann J. 2003. (E)-beta-ocimene and myrcene synthase genes of floral scent biosynthesis in snapdragon: function and expression of three terpene synthase genes of a new terpene synthase subfamily. The Plant Cell 15, 1227–1241. 12724546 10.1105/tpc.011015 PMC153728

[CIT0028] Dudareva N , Murfitt LM, Mann CJ, Gorenstein N, Kolosova N, Kish CM, Bonham C, Wood K. 2000. Developmental regulation of methyl benzoate biosynthesis and emission in snapdragon flowers. The Plant Cell 12, 949–961. 10852939 10.1105/tpc.12.6.949 PMC149095

[CIT0029] Dudareva N , Negre F. 2005. Practical applications of research into the regulation of plant volatile emission. Current Opinion in Plant Biology 8, 113–118. 15653408 10.1016/j.pbi.2004.11.007

[CIT0030] Dudareva N , Negre F, Nagegowda DA, Orlova I. 2006. Plant volatiles: recent advances and future perspectives. Critical Reviews in Plant Sciences 25, 417–440.

[CIT0031] Dudareva N , Pichersky E, Gershenzon J. 2004. Biochemistry of plant volatiles. Plant Physiology 135, 1893–1902. 15326281 10.1104/pp.104.049981 PMC520761

[CIT0032] Dutta A , Abmayr SM, Workman JL. 2016. Diverse activities of histone acylations connect metabolism to chromatin function. Molecular Cell 63, 547–552. 27540855 10.1016/j.molcel.2016.06.038 PMC5298895

[CIT0033] Effmert U , Saschenbrecker S, Ross J, Negre F, Fraser CM, Noel JP, Dudareva N, Piechulla B. 2005. Floral benzenoid carboxyl methyltransferases: from *in vitro* to in planta function. Phytochemistry 66, 1211–1230. 15946712 10.1016/j.phytochem.2005.03.031 PMC2864587

[CIT0034] Engelberth J , Alborn HT, Schmelz EA, Tumlinson JH. 2004. Airborne signals prime plants against insect herbivore attack. Proceedings of the National Academy of Sciences, USA 101, 1781–1785. 10.1073/pnas.0308037100 PMC341853 14749516

[CIT0035] Fenske MP , Hewett Hazelton KD, Hempton AK, Shim JS, Yamamoto BM, Riffell JA, Imaizumi T. 2015. Circadian clock gene LATE ELONGATED HYPOCOTYL directly regulates the timing of floral scent emission in Petunia. Proceedings of the National Academy of Sciences, USA 112, 9775–9780. 10.1073/pnas.1422875112 PMC4534231 26124104

[CIT0036] Fernandez-Pozo N , Menda N, Edwards JD, et al. 2015 *a*. The Sol Genomics Network (SGN)—from genotype to phenotype to breeding. Nucleic Acids Research 43, D1036–D1041. 25428362 10.1093/nar/gku1195 PMC4383978

[CIT0037] Fernandez-Pozo N , Rosli HG, Martin GB, Mueller LA. 2015 *b*. The SGN VIGS tool: user-friendly software to design virus-induced gene silencing (VIGS) constructs for functional genomics. Molecular Plant 8, 486–488. 25667001 10.1016/j.molp.2014.11.024

[CIT0038] Ferrer JL , Austin MB, Stewart C Jr, Noel JP. 2008. Structure and function of enzymes involved in the biosynthesis of phenylpropanoids. Plant Physiology and Biochemistry 46, 356–370. 18272377 10.1016/j.plaphy.2007.12.009 PMC2860624

[CIT0039] Fraser CM , Chapple C. 2011. The phenylpropanoid pathway in Arabidopsis. The Arabidopsis Book 9, e0152. 22303276 10.1199/tab.0152 PMC3268504

[CIT0040] Gendrel AV , Lippman Z, Martienssen R, Colot V. 2005. Profiling histone modification patterns in plants using genomic tiling microarrays. Nature Methods 2, 213–218. 16163802 10.1038/nmeth0305-213

[CIT0041] Gershenzon J , McConkey ME, Croteau RB. 2000. Regulation of monoterpene accumulation in leaves of peppermint. Plant Physiology 122, 205–214. 10631264 10.1104/pp.122.1.205 PMC58859

[CIT0042] Guterman I , Masci T, Chen X, Negre F, Pichersky E, Dudareva N, Weiss D, Vainstein A. 2006. Generation of phenylpropanoid pathway-derived volatiles in transgenic plants: rose alcohol acetyltransferase produces phenylethyl acetate and benzyl acetate in petunia flowers. Plant Molecular Biology 60, 555–563. 16525891 10.1007/s11103-005-4924-x

[CIT0043] Ha M , Ng DW, Li WH, Chen ZJ. 2011. Coordinated histone modifications are associated with gene expression variation within and between species. Genome Research 21, 590–598. 21324879 10.1101/gr.116467.110 PMC3065706

[CIT0044] He G , Elling AA, Deng XW. 2011. The epigenome and plant development. Annual Review of Plant Biology 62, 411–435. 10.1146/annurev-arplant-042110-103806 21438682

[CIT0045] Jia Y , Tian H, Li H, Yu Q, Wang L, Friml J, Ding Z. 2015. The *Arabidopsis thaliana* elongator complex subunit 2 epigenetically affects root development. Journal of Experimental Botany 66, 4631–4642. 25998905 10.1093/jxb/erv230 PMC4507768

[CIT0046] Kaur H , Shaker K, Heinzel N, Ralph J, Gális I, Baldwin IT. 2012. Environmental stresses of field growth allow cinnamyl alcohol dehydrogenase-deficient *Nicotiana attenuata* plants to compensate for their structural deficiencies. Plant Physiology 159, 1545–1570. 22645069 10.1104/pp.112.196717 PMC3425196

[CIT0047] Kessler A , Baldwin IT. 2001. Defensive function of herbivore-induced plant volatile emissions in nature. Science 291, 2141–2144. 11251117 10.1126/science.291.5511.2141

[CIT0048] Kim D , Pertea G, Trapnell C, Pimentel H, Kelley R, Salzberg SL. 2013. TopHat2: accurate alignment of transcriptomes in the presence of insertions, deletions and gene fusions. Genome Biology 14, R36. 23618408 10.1186/gb-2013-14-4-r36 PMC4053844

[CIT0049] Kim JY , Swanson RT, Alvarez MI, Johnson TS, Cho KH, Clark DG, Colquhoun TA. 2019. Down regulation of *p*-coumarate 3-hydroxylase in petunia uniquely alters the profile of emitted floral volatiles. Scientific Reports 9, 8852. 31221970 10.1038/s41598-019-45183-2 PMC6586934

[CIT0050] Koeduka T , Louie GV, Orlova I, et al. 2008. The multiple phenylpropene synthases in both *Clarkia breweri* and *Petunia hybrida* represent two distinct protein lineages. The Plant Journal 54, 362–374. 18208524 10.1111/j.1365-313X.2008.03412.x PMC2741023

[CIT0051] Kolosova N , Gorenstein N, Kish CM, Dudareva N. 2001 *b*. Regulation of circadian methyl benzoate emission in diurnally and nocturnally emitting plants. The Plant Cell 13, 2333–2347. 11595805 10.1105/tpc.010162 PMC139162

[CIT0052] Kolosova N , Sherman D, Karlson D, Dudareva N. 2001 *a*. Cellular and subcellular localization of *S*-adenosyl-l-methionine:benzoic acid carboxyl methyltransferase, the enzyme responsible for biosynthesis of the volatile ester methylbenzoate in snapdragon flowers. Plant Physiology 126, 956–964. 11457946 10.1104/pp.126.3.956 PMC116452

[CIT0053] Kost C , Heil M. 2006. Herbivore-induced plant volatiles induce an indirect defence in neighbouring plants. Journal of Ecology 94, 619–628.

[CIT0054] Krogan NJ , Greenblatt JF. 2001. Characterization of a six-subunit holo-elongator complex required for the regulated expression of a group of genes in *Saccharomyces cerevisiae*. Molecular and Cellular Biology 21, 8203–8212. 11689709 10.1128/MCB.21.23.8203-8212.2001 PMC99985

[CIT0055] Krzywinski M , Schein J, Birol I, Connors J, Gascoyne R, Horsman D, Jones SJ, Marra MA. 2009. Circos: an information aesthetic for comparative genomics. Genome Research 19, 1639–1645. 19541911 10.1101/gr.092759.109 PMC2752132

[CIT0056] Langmead B , Salzberg SL. 2012. Fast gapped-read alignment with Bowtie 2. Nature Methods 9, 357–359. 22388286 10.1038/nmeth.1923 PMC3322381

[CIT0057] Lee HG , Lee K, Jang K, Seo PJ. 2015. Circadian expression profiles of chromatin remodeling factor genes in Arabidopsis. Journal of Plant Research 128, 187–199. 25315904 10.1007/s10265-014-0665-8

[CIT0058] Li Y , Mukherjee I, Thum KE, Tanurdzic M, Katari MS, Obertello M, Edwards MB, McCombie WR, Martienssen RA, Coruzzi GM. 2015. The histone methyltransferase SDG8 mediates the epigenetic modification of light and carbon responsive genes in plants. Genome Biology 16, 79. 25928034 10.1186/s13059-015-0640-2 PMC4464704

[CIT0059] Love MI , Huber W, Anders S. 2014. Moderated estimation of fold change and dispersion for RNA-seq data with DESeq2. Genome Biology 15, 550. 25516281 10.1186/s13059-014-0550-8 PMC4302049

[CIT0060] Maeda H , Dudareva N. 2012. The shikimate pathway and aromatic amino acid biosynthesis in plants. Annual Review of Plant Biology 63, 73–105. 10.1146/annurev-arplant-042811-105439 22554242

[CIT0061] Maeda H , Shasany AK, Schnepp J, Orlova I, Taguchi G, Cooper BR, Rhodes D, Pichersky E, Dudareva N. 2010. RNAi suppression of Arogenate Dehydratase1 reveals that phenylalanine is synthesized predominantly via the arogenate pathway in petunia petals. The Plant Cell 22, 832–849. 20215586 10.1105/tpc.109.073247 PMC2861463

[CIT0062] Maeda H , Yoo H, Dudareva N. 2011. Prephenate aminotransferase directs plant phenylalanine biosynthesis via arogenate. Nature Chemical Biology 7, 19–21. 21102469 10.1038/nchembio.485

[CIT0063] Malapeira J , Khaitova LC, Mas P. 2012. Ordered changes in histone modifications at the core of the Arabidopsis circadian clock. Proceedings of the National Academy of Sciences, USA 109, 21540–21545. 10.1073/pnas.1217022110 PMC3535592 23236129

[CIT0064] Martin M . 2011. Cutadapt removes adapter sequences from high-throughput sequencing reads. EMBnet.Journal 17, 10–12.

[CIT0065] Martin DM , Gershenzon J, Bohlmann J. 2003. Induction of volatile terpene biosynthesis and diurnal emission by methyl jasmonate in foliage of Norway spruce. Plant Physiology 132, 1586–1599. 12857838 10.1104/pp.103.021196 PMC167096

[CIT0066] Muhlemann JK , Klempien A, Dudareva N. 2014 *a*. Floral volatiles: from biosynthesis to function. Plant, Cell & Environment 37, 1936–1949. 10.1111/pce.12314 24588567

[CIT0067] Muhlemann JK , Maeda H, Chang CY, et al. 2012. Developmental changes in the metabolic network of snapdragon flowers. PLoS One 7, e40381. 22808147 10.1371/journal.pone.0040381 PMC3394800

[CIT0068] Muhlemann JK , Woodworth BD, Morgan JA, Dudareva N. 2014 *b*. The monolignol pathway contributes to the biosynthesis of volatile phenylpropenes in flowers. New Phytologist 204, 661–670. 10.1111/nph.12913 24985707

[CIT0069] Murase K , Hirano Y, Sun TP, Hakoshima T. 2008. Gibberellin-induced DELLA recognition by the gibberellin receptor GID1. Nature 456, 459–463. 19037309 10.1038/nature07519

[CIT0070] Musselman CA , Lalonde ME, Côté J, Kutateladze TG. 2012. Perceiving the epigenetic landscape through histone readers. Nature Structural & Molecular Biology 19, 1218–1227. 10.1038/nsmb.2436 PMC3645987 23211769

[CIT0071] Negre F , Kish CM, Boatright J, Underwood B, Shibuya K, Wagner C, Clark DG, Dudareva N. 2003. Regulation of methylbenzoate emission after pollination in snapdragon and petunia flowers. The Plant Cell 15, 2992–3006. 14630969 10.1105/tpc.016766 PMC282847

[CIT0072] Nelissen H , De Groeve S, Fleury D, et al. 2010. Plant Elongator regulates auxin-related genes during RNA polymerase II transcription elongation. Proceedings of the National Academy of Sciences, USA 107, 1678–1683. 10.1073/pnas.0913559107 PMC2824411 20080602

[CIT0073] Nelissen H , Fleury D, Bruno L, Robles P, De Veylder L, Traas J, Micol JL, Van Montagu M, Inzé D, Van Lijsebettens M. 2005. The *elongata* mutants identify a functional Elongator complex in plants with a role in cell proliferation during organ growth. Proceedings of the National Academy of Sciences, USA 102, 7754–7759. 10.1073/pnas.0502600102 PMC1140448 15894610

[CIT0074] Oh E , Yamaguchi S, Hu J, Yusuke J, Jung B, Paik I, Lee HS, Sun TP, Kamiya Y, Choi G. 2007. PIL5, a phytochrome-interacting bHLH protein, regulates gibberellin responsiveness by binding directly to the GAI and RGA promoters in Arabidopsis seeds. The Plant Cell 19, 1192–1208. 17449805 10.1105/tpc.107.050153 PMC1913757

[CIT0075] Oliva M , Ovadia R, Perl A, Bar E, Lewinsohn E, Galili G, Oren-Shamir M. 2015. Enhanced formation of aromatic amino acids increases fragrance without affecting flower longevity or pigmentation in *Petunia* × *hybrida*. Plant Biotechnology Journal 13, 125–136. 25283446 10.1111/pbi.12253

[CIT0076] Otero G , Fellows J, Li Y, de Bizemont T, Dirac AM, Gustafsson CM, Erdjument-Bromage H, Tempst P, Svejstrup JQ. 1999. Elongator, a multisubunit component of a novel RNA polymerase II holoenzyme for transcriptional elongation. Molecular Cell 3, 109–118. 10024884 10.1016/s1097-2765(00)80179-3

[CIT0077] Pandey R , Müller A, Napoli CA, Selinger DA, Pikaard CS, Richards EJ, Bender J, Mount DW, Jorgensen RA. 2002. Analysis of histone acetyltransferase and histone deacetylase families of *Arabidopsis thaliana* suggests functional diversification of chromatin modification among multicellular eukaryotes. Nucleic Acids Research 30, 5036–5055. 12466527 10.1093/nar/gkf660 PMC137973

[CIT0078] Para A , Li Y, Coruzzi GM. 2018. μChIP-Seq for genome-wide mapping of in vivo TF–DNA interactions in Arabidopsis root protoplasts. In: Ristova D, Barbez E, eds. Root development: methods and protocols. New York: Springer New York, 249–261. 10.1007/978-1-4939-7747-5_19 29525963

[CIT0079] Passeri V , Koes R, Quattrocchio FM. 2016. New challenges for the design of high value plant products: stabilization of anthocyanins in plant vacuoles. Frontiers in Plant Science 7, 153. 26909096 10.3389/fpls.2016.00153 PMC4754442

[CIT0080] Pfannenstiel BT , Keller NP. 2019. On top of biosynthetic gene clusters: how epigenetic machinery influences secondary metabolism in fungi. Biotechnology Advances 37, 107345. 30738111 10.1016/j.biotechadv.2019.02.001 PMC6685777

[CIT0081] Plasmeijer M , Liao P, Haring MA, Schuurink RC. 2020. Metabolic engineering of plant volatiles. In: Pichersky E, Dudareva N, eds. Biology of plant volatiles, 2nd edn. Boca Raton, FL: CRC Press, 379–403.

[CIT0082] Pullman GS , Mein J, Johnson S, Zhang Y. 2005. Gibberellin inhibitors improve embryogenic tissue initiation in conifers. Plant Cell Reports 23, 596–605. 15688237 10.1007/s00299-004-0880-1

[CIT0083] Qi L , Zhang X, Zhai H, Liu J, Wu F, Li C, Chen Q. 2019. Elongator is required for root stem cell maintenance by regulating SHORTROOT transcription. Plant Physiology 179, 220–232. 30401723 10.1104/pp.18.00534 PMC6324240

[CIT0084] Qian Y , Lynch JH, Guo L, Rhodes D, Morgan JA, Dudareva N. 2019. Completion of the cytosolic post-chorismate phenylalanine biosynthetic pathway in plants. Nature Communications 10, 15. 10.1038/s41467-018-07969-2 PMC6318282 30604768

[CIT0085] Qualley AV , Dudareva N. 2008. Aromatic volatiles and their involvement in plant defense. In: Schaller A, ed. Induced plant resistance to herbivory. Dordrecht: Springer Netherlands, 409–432.

[CIT0086] Quattrocchio F , Wing JF, Leppen H, Mol J, Koes RE. 1993. Regulatory genes controlling anthocyanin pigmentation are functionally conserved among plant species and have distinct sets of target genes. The Plant Cell 5, 1497–1512. 12271045 10.1105/tpc.5.11.1497 PMC160381

[CIT0087] Quattrocchio F , Wing J, van der Woude K, Souer E, de Vetten N, Mol J, Koes R. 1999. Molecular analysis of the anthocyanin2 gene of petunia and its role in the evolution of flower color. The Plant Cell 11, 1433–1444. 10449578 10.1105/tpc.11.8.1433 PMC144295

[CIT0088] Quinlan AR , Hall IM. 2010. BEDTools: a flexible suite of utilities for comparing genomic features. Bioinformatics 26, 841–842. 20110278 10.1093/bioinformatics/btq033 PMC2832824

[CIT0089] Ravid J , Spitzer-Rimon B, Takebayashi Y, Seo M, Cna’ani A, Aravena-Calvo J, Masci T, Farhi M, Vainstein A. 2017. GA as a regulatory link between the showy floral traits color and scent. New Phytologist 215, 411–422. 10.1111/nph.14504 28262954

[CIT0090] Rea S , Eisenhaber F, O’Carroll D, et al. 2000. Regulation of chromatin structure by site-specific histone H3 methyltransferases. Nature 406, 593–599. 10949293 10.1038/35020506

[CIT0091] Reid MA , Dai Z, Locasale JW. 2017. The impact of cellular metabolism on chromatin dynamics and epigenetics. Nature Cell Biology 19, 1298–1306. 29058720 10.1038/ncb3629 PMC5886854

[CIT0092] Robinson JT , Thorvaldsdóttir H, Winckler W, Guttman M, Lander ES, Getz G, Mesirov JP. 2011. Integrative genomics viewer. Nature Biotechnology 29, 24–26. 10.1038/nbt.1754 PMC3346182 21221095

[CIT0093] Schuurink RC , Haring MA, Clark DG. 2006. Regulation of volatile benzenoid biosynthesis in petunia flowers. Trends in Plant Science 11, 20–25. 16226052 10.1016/j.tplants.2005.09.009

[CIT0094] Seabold S , Perktold J. 2010. Statsmodels: econometric and statistical modeling with Python. https://www.statsmodels.org/stable/index.html.

[CIT0095] Shaipulah NF , Muhlemann JK, Woodworth BD, Van Moerkercke A, Verdonk JC, Ramirez AA, Haring MA, Dudareva N, Schuurink RC. 2016. CCoAOMT down-regulation activates anthocyanin biosynthesis in petunia. Plant Physiology 170, 717–731. 26620524 10.1104/pp.15.01646 PMC4734575

[CIT0096] ut R , Eudes A, Mouille G, Pollet B, Lapierre C, Jouanin L, Séguin A. 2005. CINNAMYL ALCOHOL DEHYDROGENASE-C and -D are the primary genes involved in lignin biosynthesis in the floral stem of Arabidopsis. The Plant Cell 17, 2059–2076. 15937231 10.1105/tpc.105.030767 PMC1167552

[CIT0097] Spitzer-Rimon B , Marhevka E, Barkai O, Marton I, Edelbaum O, Masci T, Prathapani NK, Shklarman E, Ovadis M, Vainstein A. 2010. EOBII, a gene encoding a flower-specific regulator of phenylpropanoid volatiles’ biosynthesis in petunia. The Plant Cell 22, 1961–1976. 20543029 10.1105/tpc.109.067280 PMC2910970

[CIT0098] Strahl BD , Allis CD. 2000. The language of covalent histone modifications. Nature 403, 41–45. 10638745 10.1038/47412

[CIT0099] Strauss J , Reyes-Dominguez Y. 2011. Regulation of secondary metabolism by chromatin structure and epigenetic codes. Fungal Genetics and Biology 48, 62–69. 20659575 10.1016/j.fgb.2010.07.009 PMC3935439

[CIT0100] Supek F , Bošnjak M, Škunca N, Šmuc T. 2011. REVIGO summarizes and visualizes long lists of gene ontology terms. PLoS One 6, e21800. 21789182 10.1371/journal.pone.0021800 PMC3138752

[CIT0101] Tyler L , Thomas SG, Hu J, Dill A, Alonso JM, Ecker JR, Sun TP. 2004. Della proteins and gibberellin-regulated seed germination and floral development in Arabidopsis. Plant Physiology 135, 1008–1019. 15173565 10.1104/pp.104.039578 PMC514135

[CIT0102] Underwood BA , Tieman DM, Shibuya K, Dexter RJ, Loucas HM, Simkin AJ, Sims CA, Schmelz EA, Klee HJ, Clark DG. 2005. Ethylene-regulated floral volatile synthesis in petunia corollas. Plant Physiology 138, 255–266. 15849311 10.1104/pp.104.051144 PMC1104180

[CIT0103] Untergasser A , Cutcutache I, Koressaar T, Ye J, Faircloth BC, Remm M, Rozen SG. 2012. Primer3—new capabilities and interfaces. Nucleic Acids Research 40, e115. 22730293 10.1093/nar/gks596 PMC3424584

[CIT0104] Verdonk JC , Haring MA, van Tunen AJ, Schuurink RC. 2005. ODORANT1 regulates fragrance biosynthesis in petunia flowers. The Plant Cell 17, 1612–1624. 15805488 10.1105/tpc.104.028837 PMC1091778

[CIT0105] Verdonk JC , Ric de Vos CH, Verhoeven HA, Haring MA, van Tunen AJ, Schuurink RC. 2003. Regulation of floral scent production in petunia revealed by targeted metabolomics. Phytochemistry 62, 997–1008. 12590126 10.1016/s0031-9422(02)00707-0

[CIT0106] Vogel JT , Tan BC, McCarty DR, Klee HJ. 2008. The carotenoid cleavage dioxygenase 1 enzyme has broad substrate specificity, cleaving multiple carotenoids at two different bond positions. Journal of Biological Chemistry 283, 11364–11373. 10.1074/jbc.M710106200 18285342

[CIT0107] Widhalm JR , Gutensohn M, Yoo H, et al. 2015. Identification of a plastidial phenylalanine exporter that influences flux distribution through the phenylalanine biosynthetic network. Nature Communications 6, 8142. 10.1038/ncomms9142 PMC4647861 26356302

[CIT0108] Xu S , Grullon S, Ge K, Peng W. 2014. Spatial clustering for identification of ChIP-enriched regions (SICER) to map regions of histone methylation patterns in embryonic stem cells. Methods in Molecular Biology 1150, 97–111. 24743992 10.1007/978-1-4939-0512-6_5 PMC4152844

[CIT0109] Yamaguchi S , Kamiya Y, Sun T. 2001. Distinct cell-specific expression patterns of early and late gibberellin biosynthetic genes during Arabidopsis seed germination. The Plant Journal 28, 443–453. 11737781 10.1046/j.1365-313x.2001.01168.x

[CIT0110] Yi HS , Heil M, Adame-Alvarez RM, Ballhorn DJ, Ryu CM. 2009. Airborne induction and priming of plant defenses against a bacterial pathogen. Plant Physiology 151, 2152–2161. 19812184 10.1104/pp.109.144782 PMC2785983

[CIT0111] Yoo H , Widhalm JR, Qian Y, Maeda H, Cooper BR, Jannasch AS, Gonda I, Lewinsohn E, Rhodes D, Dudareva N. 2013. An alternative pathway contributes to phenylalanine biosynthesis in plants via a cytosolic tyrosine:phenylpyruvate aminotransferase. Nature Communications 4, 2833. 10.1038/ncomms3833 24270997

[CIT0112] Zang C , Schones DE, Zeng C, Cui K, Zhao K, Peng W. 2009. A clustering approach for identification of enriched domains from histone modification ChIP-Seq data. Bioinformatics 25, 1952–1958. 19505939 10.1093/bioinformatics/btp340 PMC2732366

[CIT0113] Zhang Y , Reinberg D. 2001. Transcription regulation by histone methylation: interplay between different covalent modifications of the core histone tails. Genes & Development 15, 2343–2360. 11562345 10.1101/gad.927301

[CIT0114] Zhao Q , Dixon RA. 2011. Transcriptional networks for lignin biosynthesis: more complex than we thought? Trends in Plant Science 16, 227–233. 21227733 10.1016/j.tplants.2010.12.005

[CIT0115] Zhao Q , Nakashima J, Chen F, Yin Y, Fu C, Yun J, Shao H, Wang X, Wang ZY, Dixon RA. 2013. Laccase is necessary and nonredundant with peroxidase for lignin polymerization during vascular development in Arabidopsis. The Plant Cell 25, 3976–3987. 24143805 10.1105/tpc.113.117770 PMC3877815

