# Supplementary material for: Dynamic histone acetylation in floral volatile synthesis and emission in petunia flowers
Source: J Exp Bot. 2021 Feb 19;72(10):3704–22. doi: 10.1093/jxb/erab072 (PMC8096599; doi:10.1093/jxb/erab072)
Supplement: erab072_suppl_Supplementary-File-1 [file erab072_suppl_supplementary-file-1.pdf]

SUPPLEMENTAL INFORMATION

Supplemental Figures

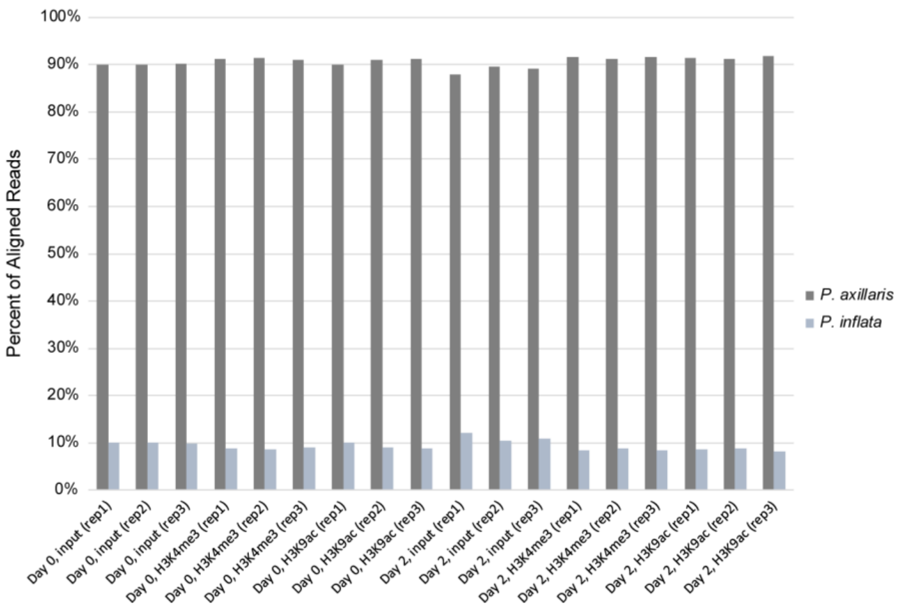

Figure S1. ChIP-Seq reads alignment to petunia genomes.
Proportions of ChIP-seq reads from *P. hybrida* cv. Mitchell that mapped to *P. axillaris* or *P. inflata* genomes are shown.

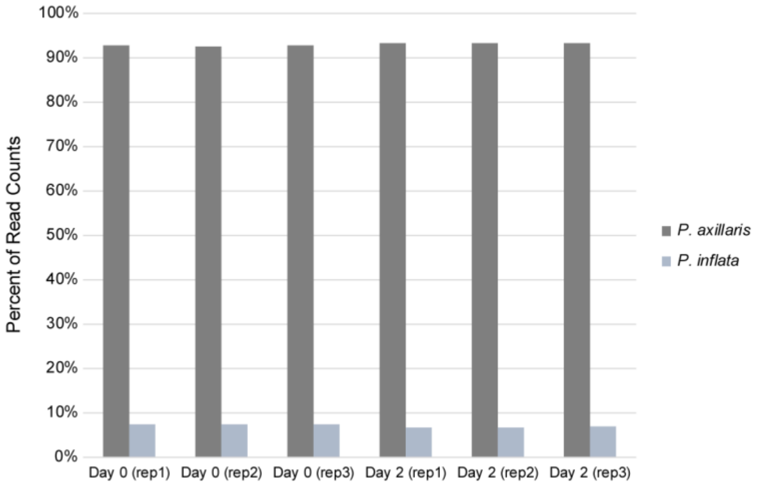

Figure S2. RNA-Seq reads alignment to petunia genomes. Proportions of RNA-seq reads from *P. hybrida* cv. Mitchell that mapped to *P. axillaris* or *P. inflata* genomes are shown.

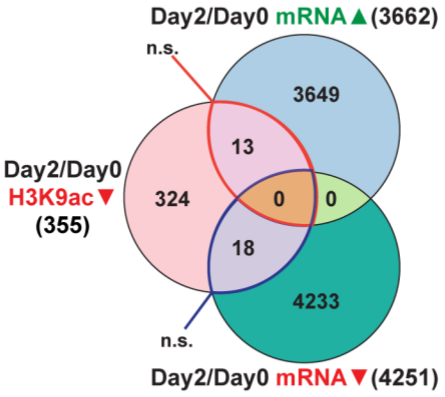

Figure S3. Intersection between DMGs with decreased H3K9ac and DEGs. No significant overlap was observed between DMGs with decreased H3K9ac and transcriptionally regulated genes.

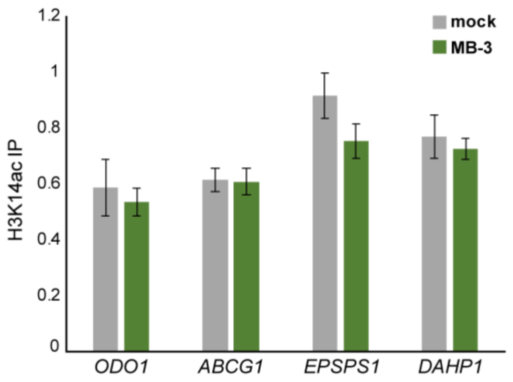

Figure S4. H3K14ac ChIP-qPCR of MB-3 treated petunia. Detached flower buds at day 0 were treated with mock (0.1% DMSO) or MB-3 (100 µM in 0.1% DMSO) for two days and then used for ChIP with antibodies against H3K14ac. qPCR was performed with gene body primers designed against predicted acetylation peaks from sequencing to determine ChIP pulldown relative to input with normalization relative to housekeeping control genes *ACTIN* and *PP2AA3* (n=4). Significance of measurements determined by Student's t-test and depicted as asterisks: $p < 0.05$ (*), $p < 0.01$ (**).

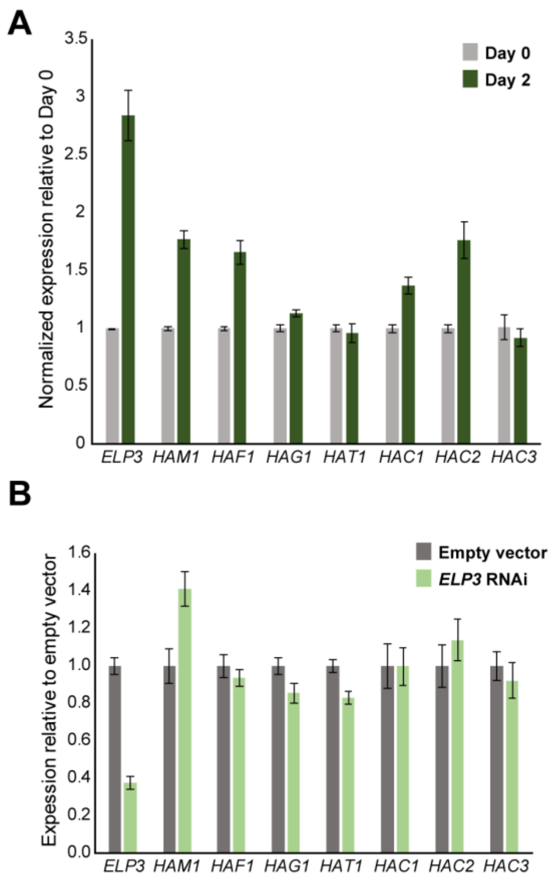

Figure S5. Expression of histone acetyltransferases (HATs) in petunia. (A) Fold-change in gene expression at Day 2 relative to Day 0 in RNA-Seq. (B) qRT-PCR of HAT genes in *ELP3* RNAi corolla relative to empty vector, with *FBP1* and *UBQ10* used as normalization controls (n=5-6).

Table S1. Primers used in the study.

Gene	Primer	Sequence (5' to 3')
FBP1	FBP1 F	ACATGCAAATTGAACTCAGGCA
FBP1	FBP1 R	GGGCATCTTCCAATATCATGAGC
ODO1	ODO1 F	GGAAATTCCCATGGCGAATCG
ODO1	ODO1 R	GCACCACTGATGAATCCAAGC
EPSPS1	EPSPS1 F	GCCTGATGTTGCCATGACAC
EPSPS1	EPSPS1 R	TGCGCTCAGTTTCCTTGACT
DAHP1	DAHP1 F	ACCATCAAAGCTCCGTGTGG
DAHP1	DAHP1 R	CTGGGTGGCTTCCTTCTTGT
CS	CS F	AGCAAACTGTGACGAGAGACA
CS	CS R	GGCTACCATTGCTTCAACCA
CNL1	CNL1 F	AGCTGTAGTTTCTTTACTTTCCCA
CNL1	CNL1 R	AAATCCAGAGATCAAAGAAGCAGA
KAT1	KAT1 F	AATGTGAATGGTGGGGCGAT
KAT1	KAT1 R	CGGCGTTTCATCTCATGCAG
CCOAOMT1	CCoAOMT1 F	TGTGGATGCTGATAAGGACAACT
CCOAOMT1	CCoAOMT1 R	CAATCAAACCACCAACCTTCACT
CFAT	CFAT F	ACGATTCCTCAACGACCATGA
CFAT	CFAT R	CTTGAGCTGTCCAACACTTGC
PP2AA3 ChIP	PP2AA3 pkF	GCACTCGGTGAGGAAAGAAC
PP2AA3 ChIP	PP2AA3 pkR	TGCAAGCAACACCTCATCAT
ACTIN ChIP	ACTIN pkF	GTATCGCGTTTCGATTGGTT
ACTIN ChIP	ACTIN pkR	AAGCGATTGACTGAGCAGCTA
ODO1 ChIP	ODO1 pkF	CGACCTGATTTGAAAAGAGGCC
ODO1 ChIP	ODO1 pkR	ACCTGTTTCCAAGACGAGAATG
ABCG1 ChIP	ABCG1 pkF	TGCTGGATCCTAGTCTCCCT
ABCG1 ChIP	ABCG1 pkr	CCAGCAGCAGTAACCAGGAA
EPSPS1 ChIP	EPSPS1 pkF	CCCTTGCCAACAACCTTCAG
EPSPS1 ChIP	EPSPS1 pkR	CTGAGTAAACCCCGCTCCTG
DAHP1 ChIP	DAHP1 pkF	AGGTGGTGATTGTGCTGAGAG
DAHP1 ChIP	DAHP1 pkR	AGGCATCTGACCACCAAACA
ELP3	ELP3 F	AATTGCTCGAAGGGAGCATA
ELP3	ELP3 R	CGGACCTTCAAGCTCATACC
HAM1	HAM1 F	TGGACTCCATACAAGGAGCA
HAM1	HAM1 R	AGTGGATCGCAGCAGCTAAT
HAF1	HAF1 F	CCAGTGACTCGAAAGGAAGC
HAF1	HAF1 R	CCGCGGTTTTTATACTCCAG
HAG1	HAG1 F	AGAGAGGAGGAATCGGGAAG
HAG1	HAG1 R	ATGTTTGGAAGTTGCCTTGC
HAT1	HAT1 F	ATGGTCCACCGTTGAGTGTT
HAT1	HAT1 R	TTGGGTCCAAGCGAAGATAG

HAC1	HAC1 F	CGCGAACGACAGTATTTCAA
HAC1	HAC1 R	CTGGACATTTCCCTTCAGGA
HAC2	HAC2 F	ACAAGCTTCTTGCCGAAGAC
HAC2	HAC2 R	CAGGTTGTTCCCACCAGAAT
HAC3	HAC3 F	GAGACCGTTGCAGGTCAAAT
HAC3	HAC3 R	CTGAATACGGGCAGGGATTA
ELP3 RNAi	ELP3 VIGS-F	GGGGACAAGTTTGTACAAAAAAGCAGGCTAAATGGCGGCGGCGGTGG
ELP3 RNAi	ELP3 VIGS-R	GGGGACCACTTTGTACAAGAAAGCTGGGTAAGTACGAACCGGTTTGGCTCGG

Table S2. Enriched GO Terms Among DMGs with increased H3K9ac.

GO Term	Description	Fold Enrichment	FDR adjusted p value
GO:0009939	positive regulation of gibberellic acid mediated signaling pathway	4.21	0.01826
GO:0018401	peptidyl-proline hydroxylation to 4-hydroxy-L-proline	4.21	0.01826
GO:0048280	vesicle fusion with Golgi apparatus	4.21	0.01826
GO:0060968	regulation of gene silencing	3.90	0.04945
GO:0009423	chorismate biosynthetic process	3.83	0.02925
GO:0090351	seedling development	3.51	3.22E-05
GO:0009073	aromatic amino acid family biosynthetic process	3.51	0.01679
GO:0090332	stomatal closure	3.16	0.01808
GO:0010093	specification of floral organ identity	3.07	0.04714
GO:0009963	positive regulation of flavonoid biosynthetic process	3.07	0.04714
GO:0000045	autophagosome assembly	2.97	0.01219
GO:0042594	response to starvation	2.81	0.02219
GO:0006914	autophagy	2.69	0.01219
GO:0006338	chromatin remodeling	2.66	0.01069
GO:0008654	phospholipid biosynthetic process	2.66	0.02219
GO:0007031	peroxisome organization	2.63	0.04714
GO:0061025	membrane fusion	2.51	0.04714
GO:0006470	protein dephosphorylation	2.47	8.97E-08
GO:0072583	clathrin-dependent endocytosis	2.46	0.0174
GO:0006396	RNA processing	2.25	0.01679
GO:0016192	vesicle-mediated transport	2.23	4.23E-06
GO:0006906	vesicle fusion	2.19	0.03911
GO:0006888	endoplasmic reticulum to Golgi vesicle-mediated transport	2.15	0.001506
GO:0009742	brassinosteroid mediated signaling pathway	2.06	0.005294
GO:0008380	RNA splicing	2.02	0.000782
GO:0006886	intracellular protein transport	1.92	6.12E-06
GO:0006364	rRNA processing	1.90	0.0109
GO:0018105	peptidyl-serine phosphorylation	1.85	0.0253
GO:0006970	response to osmotic stress	1.81	0.002575
GO:0006511	ubiquitin-dependent protein catabolic process	1.78	1.51E-06
GO:0010468	regulation of gene expression	1.78	0.04714
GO:0035556	intracellular signal transduction	1.76	0.006288
GO:0006397	mRNA processing	1.72	0.002424
GO:0000398	mRNA splicing, via spliceosome	1.69	0.02027
GO:0046777	protein autophosphorylation	1.64	0.002575
GO:0009845	seed germination	1.64	0.03157
GO:0015031	protein transport	1.53	0.01219
GO:0006412	Translation	1.37	0.0365

Table S3. Enriched GO Terms among DMGs with increased H3K4me3.

GO Term	Description	Fold Enrichment	FDR adjusted p value
GO:0010072	primary shoot apical meristem specification	8.42	0.007278
GO:0009933	meristem structural organization	6.73	0.007278
GO:0046854	phosphatidylinositol phosphorylation	5.94	0.007278
GO:0030433	ubiquitin-dependent ERAD pathway	5.05	0.01191
GO:0007018	microtubule-based movement	3.99	0.007278
GO:0048481	plant ovule development	3.99	0.0158
GO:0010305	leaf vascular tissue pattern formation	3.91	0.03853
GO:0010090	trichome morphogenesis	3.88	0.02942
GO:0010286	heat acclimation	3.81	0.02942
GO:0000209	protein polyubiquitination	3.74	0.02942
GO:0009630	gravitropism	3.67	0.02942
GO:0006364	rRNA processing	3.15	0.01305
GO:0016192	vesicle-mediated transport	3.12	0.007278
GO:0008380	RNA splicing	3.06	0.007635
GO:0018105	peptidyl-serine phosphorylation	2.96	0.02942
GO:0007049	cell cycle	2.74	0.02811
GO:0051301	cell division	2.22	0.007635
GO:0006886	intracellular protein transport	2.06	0.03345
GO:0009734	auxin-activated signaling pathway	2.04	0.03547
GO:0009793	embryo development ending in seed dormancy	1.87	0.007278
GO:0042742	defense response to bacterium	1.85	0.02178
GO:0006468	protein phosphorylation	1.48	0.02942

Table S4. Enriched GO terms among upregulated (A) or down-regulated (B) DEGs.

**(A). Enriched GO Terms Among Transcriptionally Increased DEGs**			
**GO Term**	**Description**	**Fold Enrichment**	**FDR adjusted p value**
GO:0006267	pre-replicative complex assembly involved in nuclear cell cycle DNA replication	5.12	0.002339
GO:0009423	chorismate biosynthetic process	4.89	0.000161
GO:0030308	negative regulation of cell growth	4.78	0.000647
GO:0009939	positive regulation of gibberellic acid mediated signaling pathway	4.65	0.001802
GO:1900424	regulation of defense response to bacterium	4.27	0.01546
GO:0006556	S-adenosylmethionine biosynthetic process	4.27	0.01546
GO:0060866	leaf abscission	4.27	0.01546
GO:2000762	regulation of phenylpropanoid metabolic process	3.84	0.001799
GO:0009073	aromatic amino acid family biosynthetic process	3.73	0.0013
GO:0009759	indole glucosinolate biosynthetic process	3.73	0.02699
GO:0009699	phenylpropanoid biosynthetic process	3.68	0.004775
GO:0010325	raffinose family oligosaccharide biosynthetic process	3.58	0.01671
GO:0045487	gibberellin catabolic process	3.58	0.01671
GO:0006817	phosphate ion transport	3.48	0.01148
GO:0010120	camalexin biosynthetic process	3.48	0.01148
GO:0080092	regulation of pollen tube growth	3.39	5.70E-05
GO:0006885	regulation of pH	3.36	0.004788
GO:0009682	induced systemic resistance	3.26	0.02699
GO:1900056	negative regulation of leaf senescence	3.22	0.01671
GO:0015770	sucrose transport	3.22	0.01671
GO:0000727	double-strand break repair via break-induced replication	3.22	0.01671
GO:0008283	cell population proliferation	3.14	0.004775
GO:0009051	pentose-phosphate shunt, oxidative branch	2.99	0.03835
GO:0010411	xyloglucan metabolic process	2.91	0.000251
GO:0006270	DNA replication initiation	2.81	0.0218
GO:0009864	induced systemic resistance, jasmonic acid mediated signaling pathway	2.81	0.0218
GO:0048544	recognition of pollen	2.80	0.001484
GO:0009625	response to insect	2.72	0.01292
GO:0042546	cell wall biogenesis	2.64	0.000666
GO:1900150	regulation of defense response to fungus	2.56	0.02582
GO:0009750	response to fructose	2.53	0.005202
GO:0006730	one-carbon metabolic process	2.53	0.01515
GO:0009407	toxin catabolic process	2.48	0.002674
GO:0045490	pectin catabolic process	2.44	0.000161
GO:0009741	response to brassinosteroid	2.44	0.00132
GO:0050829	defense response to Gram-negative bacterium	2.39	0.02737
GO:0061408	positive regulation of transcription from RNA polymerase II promoter in response to heat stress	2.34	0.04075

GO:0051260	protein homooligomerization	2.30	0.03428
GO:0006749	glutathione metabolic process	2.28	0.007909
GO:0010583	response to cyclopentenone	2.24	0.005202
GO:0006995	cellular response to nitrogen starvation	2.21	0.04167
GO:0010431	seed maturation	2.19	0.03428
GO:0009686	gibberellin biosynthetic process	2.19	0.03428
GO:0000160	phosphorelay signal transduction system	2.16	0.02422
GO:0002229	defense response to oomycetes	2.15	0.007909
GO:0010200	response to chitin	2.13	4.96E-05
GO:0000302	response to reactive oxygen species	2.13	0.004775
GO:0042545	cell wall modification	2.11	0.009612
GO:0006813	potassium ion transport	2.10	0.007909
GO:0019760	glucosinolate metabolic process	2.04	0.02775
GO:0009873	ethylene-activated signaling pathway	1.96	2.47E-05
GO:0042542	response to hydrogen peroxide	1.94	0.004987
GO:0010118	stomatal movement	1.94	0.04906
GO:0009860	pollen tube growth	1.86	0.001369
GO:0009809	lignin biosynthetic process	1.85	0.007745
GO:0016036	cellular response to phosphate starvation	1.77	0.01788
GO:0009751	response to salicylic acid	1.71	0.003832
GO:0010119	regulation of stomatal movement	1.70	0.03122
GO:0006351	transcription, DNA-templated	1.70	0.03945
GO:0051603	proteolysis involved in cellular protein catabolic process	1.69	0.03696
GO:0009611	response to wounding	1.67	1.61E-04
GO:0006468	protein phosphorylation	1.60	5.89E-10
GO:0007165	signal transduction	1.60	0.003052
GO:0050832	defense response to fungus	1.57	0.003947
GO:0080167	response to karrikin	1.56	0.01292
GO:0046777	protein autophosphorylation	1.54	0.005947
GO:0035556	intracellular signal transduction	1.54	2.74E-02
GO:0006970	response to osmotic stress	1.52	0.03434
GO:0006952	defense response	1.49	0.000161
GO:0009414	response to water deprivation	1.49	0.001154
GO:0009753	response to jasmonic acid	1.48	0.02737
GO:0009738	abscisic acid-activated signaling pathway	1.47	0.009612
GO:0006355	regulation of transcription, DNA-templated	1.45	2.41E-09
GO:0009734	auxin-activated signaling pathway	1.43	0.03037
GO:0055085	transmembrane transport	1.39	0.01559
GO:0006979	response to oxidative stress	1.35	0.03222
GO:0009737	response to abscisic acid	1.30	0.0184
GO:0016567	protein ubiquitination	1.27	0.01788
GO:0055114	oxidation-reduction process	1.20	0.009469

(**B). Enriched GO Terms Among Transcriptionally Decreased DEGs**			
GO:0071491	cellular response to red light	5.06	8.11E-05
GO:0071490	cellular response to far red light	5.06	8.11E-05
GO:0010608	posttranscriptional regulation of gene expression	5.06	0.004366
GO:0006696	ergosterol biosynthetic process	4.34	0.004689
GO:0010143	cutin biosynthetic process	4.05	0.001355
GO:0071492	cellular response to UV-A	4.05	0.001355
GO:0042372	phylloquinone biosynthetic process	4.05	0.001355
GO:0071483	cellular response to blue light	3.68	0.003341
GO:0010440	stomatal lineage progression	3.61	0.03573
GO:0019388	galactose catabolic process	3.61	0.03573
GO:0009638	phototropism	3.52	1.45E-05
GO:0045493	xylan catabolic process	3.46	0.000181
GO:0031222	arabinan catabolic process	3.37	0.001849
GO:0071486	cellular response to high light intensity	3.37	0.006775
GO:0046355	mannan catabolic process	3.22	0.02127
GO:0030245	cellulose catabolic process	3.04	0.008814
GO:0009854	oxidative photosynthetic carbon pathway	3.04	0.0419
GO:0009902	chloroplast relocation	3.04	0.0419
GO:0035336	long-chain fatty-acyl-CoA metabolic process	2.95	0.03291
GO:0010117	photoprotection	2.89	0.002992
GO:0032544	plastid translation	2.89	0.02377
GO:0009099	valine biosynthetic process	2.89	0.02377
GO:0002181	cytoplasmic translation	2.88	4.88E-06
GO:0042335	cuticle development	2.81	6.85E-06
GO:0009082	branched-chain amino acid biosynthetic process	2.72	0.04287
GO:0006833	water transport	2.70	0.001095
GO:0009773	photosynthetic electron transport in photosystem I	2.70	0.03291
GO:0010206	photosystem II repair	2.70	0.03291
GO:0009834	plant-type secondary cell wall biogenesis	2.69	6.62E-07
GO:0009098	leucine biosynthetic process	2.68	0.02478
GO:0016126	sterol biosynthetic process	2.53	0.000464
GO:0010207	photosystem II assembly	2.53	0.004546
GO:0009969	xyloglucan biosynthetic process	2.53	0.04287
GO:0007010	cytoskeleton organization	2.53	0.04287
GO:0010025	wax biosynthetic process	2.45	0.003135
GO:0045492	xylan biosynthetic process	2.45	0.003135
GO:0009718	anthocyanin-containing compound biosynthetic process	2.41	0.03291
GO:0006412	translation	2.33	1.65E-23
GO:0007018	microtubule-based movement	2.33	8.11E-05
GO:0000027	ribosomal large subunit assembly	2.28	0.003856
GO:0009828	plant-type cell wall loosening	2.25	0.02959

GO:0007017	microtubule-based process	2.12	0.03291
GO:0000028	ribosomal small subunit assembly	2.09	0.04287
GO:0045490	pectin catabolic process	2.07	0.001355
GO:0016042	lipid catabolic process	2.03	8.78E-06
GO:0009636	response to toxic substance	2.02	0.02627
GO:0009832	plant-type cell wall biogenesis	1.96	0.0288
GO:0015979	photosynthesis	1.95	0.000234
GO:0045489	pectin biosynthetic process	1.93	0.03613
GO:0042545	cell wall modification	1.89	0.02803
GO:0009664	plant-type cell wall organization	1.87	0.03847
GO:0009813	flavonoid biosynthetic process	1.82	0.04019
GO:0005975	carbohydrate metabolic process	1.79	4.88E-06
GO:0071555	cell wall organization	1.74	4.88E-06
GO:0080167	response to karrikin	1.72	0.000351
GO:0009809	lignin biosynthetic process	1.69	0.02248
GO:0042744	hydrogen peroxide catabolic process	1.69	0.03291
GO:0032259	methylation	1.63	0.001155
GO:0010119	regulation of stomatal movement	1.62	0.0419
GO:0006629	lipid metabolic process	1.61	0.03845
GO:0009826	unidimensional cell growth	1.60	0.01337
GO:0009735	response to cytokinin	1.43	0.005206
GO:0055114	oxidation-reduction process	1.37	1.67E-08
GO:0055085	transmembrane transport	1.31	0.03993

Table S5. Genes involved in Shikimate and Phenylalanine Synthesis (A), General Phenylpropanoid Pathway (B) or Volatile Benzenoid and Phenylpropanoid Synthesis Genes (C).

(A) Genes involved in Shikimate and Phenylalanine Synthesis			
**Gene ID**	**Name**	**UniProt BLAST Homolog**	**E Value**
Peaxi162Scf00959g00022	EPSPS1	AROA_PETHY	0
Peaxi162Scf00013g03114	EPSPS2	A0A1S4C0K4_TOBAC	0
Peaxi162Scf00328g00523	DHQS	A0A1S3ZK76_TOBAC	0
Peaxi162Scf01039g00134	DHD-SDH1	A0A1S3YVF3_TOBAC	0
Peaxi162Scf01067g00113	DHD-SDH2	A0A1J6I158_NICAT	0
Peaxi162Scf00780g00005	DHD-SDH3	Q6PUG0_TOBAC	0
Peaxi162Scf00359g00118	SK	M1BDG7_SOLTU	0
Peaxi162Scf00030g01715	DAHP1	A0A067XH53_PETHY	0
Peaxi162Scf01060g00145	DAHP2	A0A067XGX8_PETHY	0
Peaxi162Scf00381g00086	DAHP3	A0A1U7VXQ8_NICSY	0
Peaxi162Scf00747g00122	CS	M1C9E1_SOLTU	0
Peaxi162Scf00166g00931	CM1	D2CSU4_PETHY	3.4E-176
Peaxi162Scf00495g00010	CM2	D2CSU5_PETHY	0
Peaxi162Scf00183g01421	PPA-AT	PAT_PETHY	0
Peaxi162Scf00027g00165	PPY-AT1	V5M241_PETHY	0
Peaxi162Scf00059g00920	PPY-AT2	A0A1U7VA27_NICSY	0
Peaxi162Scf00876g10023	PPY-AT3	A0A314L8Z5_NICAT	0
Peaxi162Scf00845g00027	PPY-AT4	A0A0D3LRD5_ATRBE	0
Peaxi162Scf00845g00218	PPY-AT5	A0A1J6IPT9_NICAT	0
Peaxi162Scf00114g00001	ADT1	D3U715_PETHY	0
Peaxi162Scf00389g00425	ADT2	D3U716_PETHY	0
Peaxi162Scf00002g00514	ADT3	D3U717_PETHY	0
Peaxi162Scf00147g00613	ADT4	A0A1U7URI8_NICSY	0
Peaxi162Scf00047g01817	ADT5	A0A2R6QWM5_ACTCH	5.9E-133
**(B) Genes involved in General Phenylpropanoid Pathway**			
**Gene ID**	**Name**	**UniProt BLAST Homology**	**E Value**
Peaxi162Scf00745g00865	4CL-like	A0A1J6IU55_NICAT	0
Peaxi162Scf00858g00215	PALa	Previously Annotated	
Peaxi162Scf00488g00074	PALb	Previously Annotated	
Peaxi162Scf00123g00096	PALc	Previously Annotated	
Peaxi162Scf00556g00035	C4Ha	Previously Annotated	
Peaxi162Scf00390g00225	C4Hb	Previously Annotated	
Peaxi162Scf00314g00086	4CLa	Previously Annotated	
Peaxi162Scf00195g01223	4CLb	Previously Annotated	
Peaxi162Scf00610g00346	4CLc	Previously Annotated	
Peaxi162Scf00207g00334	4CLd	Previously Annotated	
**(C) Genes involved in Volatile Benzenoid and Phenylpropanoid Synthesis**			
**Gene ID**	**Name**	**UniProt BLAST Homology**	**E Value**

Peaxi162Scf00017g02715	BALDH1	A0A314L456_NICAT	0
Peaxi162Scf00393g00118	BALDH2	A0A1J6IE01_NICAT	0
Peaxi162Scf00047g01234	KAT2	A0A1J6JQC6_NICAT	0
Peaxi162Scf00294g00211	CNL2b	J9Q6B2_PETHY	4.2E-125
Peaxi162Scf00007g00012	BPBT1	Previously Annotated	
Peaxi162Scf00007g00011	BPBT2	Previously Annotated	
Peaxi162Scf00047g01123	BSMT1	Previously Annotated	
Peaxi162Scf00047g00116	BSMT2	Previously Annotated	
Peaxi162Scf00047g01129	BSMT3	Previously Annotated	
Peaxi162Scf03967g00005	BSMT4	Previously Annotated	
Peaxi162Scf00423g00119	BSMT6	Previously Annotated	
Peaxi162Scf00231g00330	CHD1	Previously Annotated	
Peaxi162Scf01363g00003	CHD2	Previously Annotated	
Peaxi162Scf00294g00411	CNL1	Previously Annotated	
Peaxi162Scf00784g00010	CNL2	Previously Annotated	
Peaxi162Scf00294g00032	CNL3	Previously Annotated	
Peaxi162Scf00052g00819	KAT1	Previously Annotated	
Peaxi162Scf00561g00021	PAAS1	Previously Annotated	
Peaxi162Scf00152g00237	PAAS3	Previously Annotated	

Table S6. Flavonoid and Anthocyanin Synthesis Genes.

**Gene ID**	**Name**	**UniProt BLAST Homology**	**E Value**
Peaxi162Scf00817g00216	CHS-F	CHSF_PETHY	0
Peaxi162Scf00830g00016	CHS-G2	CHSG_PETHY	0
Peaxi162Scf00830g00048	CHS-G1	CHSG_PETHY	0
**Previously Annotated**			
**Gene ID**	**Name**		
Peaxi162Scf00047g01225	CHS-A		
Peaxi162Scf00536g00092	CHS-J		
Peaxi162Scf00006g00088	CHI-A		
Peaxi162Scf00038g01957	CHI-B		
Peaxi162Scf00927g00035	FLS		
Peaxi162Scf00328g01214	F3H		
Peaxi162Scf00150g00218	F3'5'H1		
Peaxi162Scf00108g00417	F3'5'H2		
Peaxi162Scf00201g00243	F3'H		
Peaxi162Scf00366g00630	DFR		
Peaxi162Scf00620g00533	ANS		
Peaxi162Scf00163g00081	3GT		
Peaxi162Scf00487g00064	RT		
Peaxi162Scf00378g00113	5GT		
Peaxi162Scf00518g00430	MT		
Peaxi162Scf00316g00055	MF		
Peaxi162Scf00713g00038	AN9		

Table S7. Genes involved in monolignol pathway, eugenol/isoeugenol synthesis, or lignin Polymerization.

Gene ID	Name	UniProt BLAST Homology	E Value
Peaxi162Scf00016g02329	CAD1	CADH1_TOBAC	0
Peaxi162Scf00152g00245	CAD2	A0A1U7X9D0_NICSY	0
Peaxi162Scf00003g02440	CCoAOMT1	A0A0S2UWC9_PETHY	2.2E-154
Peaxi162Scf00450g00032	CCoAOMT2	A0A2U1N075_ARTAN	0
Peaxi162Scf00016g02023	CCoAOMT3	A0A0S2UWA5_PETHY	1.1E-180
Peaxi162Scf00207g00444	CCR1	A0A059TC02_PETHY	0
Peaxi162Scf00332g00433	CCR2	A0A2G2WQR4_CAPBA	0
Peaxi162Scf00220g00211	C3H	A0A2L0WT18_PETHY	0
Peaxi162Scf00553g00538	HCT1	A0A2L0WT29_PETHY	0
Peaxi162Scf00835g00312	HCT2	HST_TOBAC	0
Peaxi162Scf00034g00114	CSE	A0A2U8RMZ3_PETHY	0
Peaxi162Scf00774g00314	LAC2	AT2G29130	0
Peaxi162Scf00009g01927	LAC4a	AT2G38080	0
Peaxi162Scf00141g00720	LAC4b	AT2G38080	0
Peaxi162Scf01058g00155	LAC4c	AT2G38080	0
Peaxi162Scf00366g00528	LAC11a	AT5G03260	0
Peaxi162Scf00366g00529	LAC11b	AT5G03260	0
Peaxi162Scf00226g00919	LAC14	AT5G09360	0
Peaxi162Scf00160g01641	LAC15	AT5G48100	2.2E-154
Peaxi162Scf00094g01013	LAC17a	AT5G60020	0
Peaxi162Scf00165g00079	LAC17b	AT5G60020	0
Peaxi162Scf00561g00005	LAC17c	AT5G60020	0
Peaxi162Scf00561g00008	LAC17d	AT5G60020	3.9E-167
Peaxi162Scf00561g00211	LAC17e	AT5G60020	0
Peaxi162Scf00328g00220	PER64a	AT5G42180	9.9E-145
Peaxi162Scf00328g00314	PER64b	AT5G42180	1.9E-140
Peaxi162Scf00542g00313	PER64c	AT5G42180	5.81E-91
Peaxi162Scf00192g00522	PRX66	AT5G51890	1.4E-174
Peaxi162Scf00418g00927	PRX72	AT5G66390	1.2E-180
**Previously Annotated**			
**Gene ID**	**Name**		
Peaxi162Scf00474g00217	CFAT		
Peaxi162Scf00020g01714	EGS		
Peaxi162Scf00889g00229	IGS1		
Peaxi162Scf00060g00021	IGS2		

Table S8. Histone Acetyltransferase Genes.

**Gene ID**	**Family**	**Name**	***A. thaliana* BLAST Homology**	**E Value**
Peaxi162Scf00097g01412	P300/CBP	HAC1	AT1G16710	0
Peaxi162Scf00329g00813	P300/CBP	HAC2	AT1G16710	0
Peaxi162Scf01056g00249	P300/CBP	HAC3	AT1G79000	0
Peaxi162Scf00089g00242	GNAT	ELP3	AT5G50320	4.41E-89
Peaxi162Scf01468g00004	GNAT	HAT1	AT5G56740	1.08E-176
Peaxi162Scf01826g00015	GNAT	HAG1	AT3G54610	1E-166
Peaxi162Scf00360g00314	MYST	HAM1	AT5G64610	0
Peaxi162Scf00610g00377	TAF II 250	HAF1	AT1G32750	0

Table S9. Gibberellic Acid Signaling and Metabolism Genes.

**Gene ID**	**Name**	***A. thaliana* BLAST Homology**	**E Value**
Peaxi162Scf00361g00101	GAI	AT1G14920	2.33E-156
Peaxi162Scf00305g00129	RGA1a	AT2G01570	0
Peaxi162Scf00159g00167	RGA1b	AT2G01570	0
Peaxi162Scf00052g01817	PIL5	AT2G20180	2.42E-78
Peaxi162Scf01039g00226	GID1B1	AT3G63010	0
Peaxi162Scf00006g00389	GID1B2	AT3G63010	0
Peaxi162Scf00936g00034	GID1B3	AT3G63010	0
Peaxi162Scf00434g00079	GID1C	AT5G27320	0
Peaxi162Scf01514g00021	KO1	AT5G25900	0
Peaxi162Scf01110g00313	KAO1	AT1G05160	0
Peaxi162Scf00312g00310	KAO2a	AT2G32440	1.75E-117
Peaxi162Scf00312g00313	KAO2b	AT2G32440	2.37E-103
Peaxi162Scf00312g00017	KAO2c	AT2G32440	5.27E-115
Peaxi162Scf01178g00015	GA20ox1	AT4G25420	3.07E-177
Peaxi162Scf00035g00149	GA2ox1	AT1G78440	9.82E-130
Peaxi162Scf00111g00922	GA2ox2a	AT1G30040	4.88E-136
Peaxi162Scf00111g00923	GA2ox2b	AT1G30040	2.5E-138
